# Pharmaceutical Quality by Design Approach to Develop High-Performance Nanoparticles for Magnetic Hyperthermia

**DOI:** 10.1021/acsnano.4c04685

**Published:** 2024-05-30

**Authors:** Shaquib Rahman Ansari, Yael del Carmen Suárez-López, Thomas Thersleff, Lennart Häggström, Tore Ericsson, Ioannis Katsaros, Michelle Åhlén, Maria Karlgren, Peter Svedlindh, Carlos M. Rinaldi-Ramos, Alexandra Teleki

**Affiliations:** † Department of Pharmacy, Science for Life Laboratory, Uppsala University, 75123 Uppsala, Sweden; ‡ Department of Materials and Environmental Chemistry, Stockholm University, 10691 Stockholm, Sweden; § Department of Physics and Astronomy, Uppsala University, 75121 Uppsala, Sweden; ∥ Department of Materials Science and Engineering, Uppsala University, 75103 Uppsala, Sweden; ⊥ Department of Pharmacy, Uppsala University, 75123 Uppsala, Sweden; # Department of Chemical Engineering and J. Crayton Pruitt Family Department of Biomedical Engineering, University of Florida, Gainesville, Florida 32611-6005, United States

**Keywords:** quality by design, superparamagnetic nanoparticles, magnetic hyperthermia, design of experiments, flame spray pyrolysis, doped ferrites

## Abstract

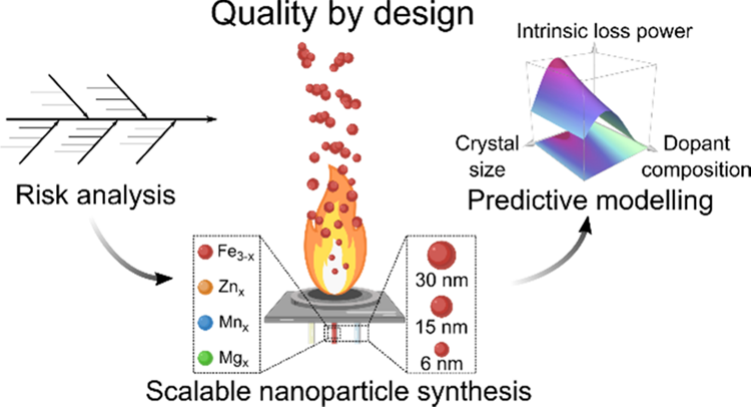

Magnetic hyperthermia holds significant therapeutic potential, yet its clinical adoption faces challenges. One obstacle is the large-scale synthesis of high-quality superparamagnetic iron oxide nanoparticles (SPIONs) required for inducing hyperthermia. Robust and scalable manufacturing would ensure control over the key quality attributes of SPIONs, and facilitate clinical translation and regulatory approval. Therefore, we implemented a risk-based pharmaceutical quality by design (QbD) approach for SPION production using flame spray pyrolysis (FSP), a scalable technique with excellent batch-to-batch consistency. A design of experiments method enabled precise size control during manufacturing. Subsequent modeling linked the SPION size (6–30 nm) and composition to intrinsic loss power (ILP), a measure of hyperthermia performance. FSP successfully fine-tuned the SPION composition with dopants (Zn, Mn, Mg), at various concentrations. Hyperthermia performance showed a strong nonlinear relationship with SPION size and composition. Moreover, the ILP demonstrated a stronger correlation to coercivity and remanence than to the saturation magnetization of SPIONs. The optimal operating space identified the midsized (15–18 nm) Mn_0.25_Fe_2.75_O_4_ as the most promising nanoparticle for hyperthermia. The production of these nanoparticles on a pilot scale showed the feasibility of large-scale manufacturing, and cytotoxicity investigations in multiple cell lines confirmed their biocompatibility. *In vitr*o hyperthermia studies with Caco-2 cells revealed that Mn_0.25_Fe_2.75_O_4_ nanoparticles induced 80% greater cell death than undoped SPIONs. The systematic QbD approach developed here incorporates process robustness, scalability, and predictability, thus, supporting the clinical translation of high-performance SPIONs for magnetic hyperthermia.

One of the most challenging steps in the clinical translation of nanomedicine is scaling up manufacturing processes to industrial volumes while maintaining product quality.^1,2^ Magnetic hyperthermia is one such clinical nanotherapeutic facing difficulties in wider implementation. The technique relies on heat generated by superparamagnetic iron oxide nanoparticles (SPIONs) in an alternating magnetic field (AMF) to induce apoptosis of tumor cells by elevating the temperature in the tumor environment to 42–46 °C.^3,4^ It is also used in antimicrobial therapy, triggered drug delivery, and image-guided therapy owing to the ability of SPIONs to serve as contrast agents in magnetic resonance imaging (MRI) and magnetic particle imaging.^5,6^ While the US Food and Drug Administration (FDA) has approved the use of SPIONs as MRI contrast agent (Ferumoxsil) and anemia treatment (Ferumoxytol), clinical translation of magnetic hyperthermia remains solely limited to the NanoTherm therapy developed by MagForce AG.^7^ Challenges related to SPIONs such as batch reproducibility, clinical efficacy, and regulatory hurdles hinder the wider clinical adoption of magnetic hyperthermia.^5^ Current industrial production methods have a limited ability to synthesize the nanoparticles on a large scale with batch-to-batch consistency.^8−10^ A quality management method can overcome this challenge by performing a systematic risk assessment of a scalable synthesis method, with validated and reproducible protocols specific to SPIONs.

SPIONs primarily consist of maghemite (γ-Fe_2_O_3_) or magnetite (Fe_3_O_4_). However, an increasing number of studies (>25%) use mixed ferrites, i.e., SPIONs doped with cobalt, zinc, manganese, nickel, or magnesium, to enhance their performance in various biomedical applications.^11^ SPIONs are typically smaller than 25–30 nm, magnetized only under an external magnetic field, and show high magnetic susceptibility, low cytotoxicity, and efficient clearance from the body, making them suitable for clinical use.^12^ The heat dissipation from SPIONs in an AMF depends on multiple physical and magnetic properties, including particle size, shape, size distribution, and magnetic anisotropy.^7^ External factors such as amplitude and frequency of the applied AMF, viscosity of the medium, and nanoparticle concentration also play a role.^13^ Clinical viability of the formulation often imposes restrictions on these external factors, necessitating modification of the SPION properties to enhance hyperthermia performance. Thus, controlling the physical and magnetic properties of SPIONs and understanding their impact on hyperthermia performance are paramount for extending their use in biomedical applications.

SPIONs are commonly produced using chemical synthesis routes like thermal decomposition and coprecipitation (Table S1). Thermal decomposition allows precise control of size and shape to produce highly crystalline particles with a narrow size distribution. However, this method is hindered by its complexity, long processing time, and low scalability. Conversely, coprecipitation is a simple method with short processing time and high particle yield, but it exhibits modest particle crystallinity, relatively large size distribution, and limited control over particle size.^14,15^ Scaling up either of these processes introduces challenges related to process complexity, product yield, and fine control of size and size distribution.^9,16^ As an example, the treatment of glioblastoma with magnetic hyperthermia therapy uses 5–12 mL of SPION suspension, with an iron concentration of 112 g_Fe_ L^–1^, corresponding to an iron oxide nanoparticle dose of up to 1.9 g for a single treatment.^17^ Thus, the quantity of nanoparticles required for treatment of a large population is difficult to attain with milligram-scale synthesis methods. Clinical translation necessitates the development of a robust large-scale synthesis method capable of producing high-quality SPIONs.

One rapid and scalable aerosol process is flame spray pyrolysis (FSP). It is capable of producing up to 12.5 kg h^–1^ nanoparticles on a pilot scale. This is equivalent to a production rate of up to 2000 doses of SPIONs per hour.^18^ The versatility of FSP in synthesizing highly pure SPIONs with reproducible control over particle size has been extensively demonstrated.^19−21^ FSP also enables the control of SPION composition by doping with metals, achieved by careful selection of the precursor salts, solvents, and the application of fundamental techniques in chemistry.^22,23^ Recent advances in theoretical and experimental investigations of FSP have significantly improved our understanding of the empirical laws governing it. However, studies investigating the effects of FSP parameters on nanoparticle properties mainly use the one-factor-at-a-time method. This approach does not provide a systematic understanding of the interaction effects between FSP parameters, the relative magnitude of the effects, or the process predictability. Systematic multivariate analysis of magnetic hyperthermia is sparsely reported.^24^ Furthermore, the FSP technique has not been explored in the pharmaceutical industry, necessitating a thorough translational study based on pharmaceutical principles. Therefore, a pre-emptive and comprehensive consideration of the chemistry, manufacturing, and control aspects of the nanomedicine development at an early stage is crucial for a successful translation. This can be achieved through the quality by design (QbD) approach.

QbD is a systematic approach to drug product development that emphasizes the understanding and control of the product and process by using sound science and risk management. The FDA and the European Medicines Agency encourage the use of risk-based approaches and QbD principles in drug product development and manufacturing.^25^ This is further emphasized in the guidelines by the International Council for Harmonization of Technical Requirements for Pharmaceuticals for Human Use (ICH Q8 and ICH Q9).^26,27^ The QbD process involves defining the quality target product profile (QTPP) and critical quality attributes (CQAs) based on risk assessment. This is followed by identifying critical process parameters (CPPs) of the manufacturing method affecting the CQAs, and executing the design of experiments (DoE) to investigate the relationship between the CQAs and the CPPs. The QbD approach facilitates in-process control, monitors quality attributes, accounts for process deviations, and ensures that the final formulation meets the specifications. It is widely used by global pharmaceutical companies, such as AstraZeneca and Pfizer, because it provides comprehensive information with minimum experiments.^28^

In this study, we demonstrate that the QbD approach of implementing risk assessment with DoE is a valuable tool to systematically investigate the effect of SPION particle properties on their magnetic hyperthermia performance, in the context of FSP synthesis (Scheme 1). Based on the risk analysis, we defined the CQAs and the critical FSP factors that can affect the CQAs. A systematic DoE approach was used to quantify and link the effects of nanoparticle size (6–30 nm), dopants (Zn, Mn, and Mg), and dopant concentration on the magnetic hyperthermia performance of SPIONs. The chemical, structural, morphological, magnetic, and heating properties of SPIONs were assessed, and a process design space was created that met all the QTPP criteria for magnetic hyperthermia. Finally, the optimal ferrite nanoparticle was investigated for *in vitro* magnetic hyperthermia. In summary, our study reports a systematic industrial approach to support and boost the clinical translation of SPIONs for magnetic hyperthermia.

**Scheme 1:**
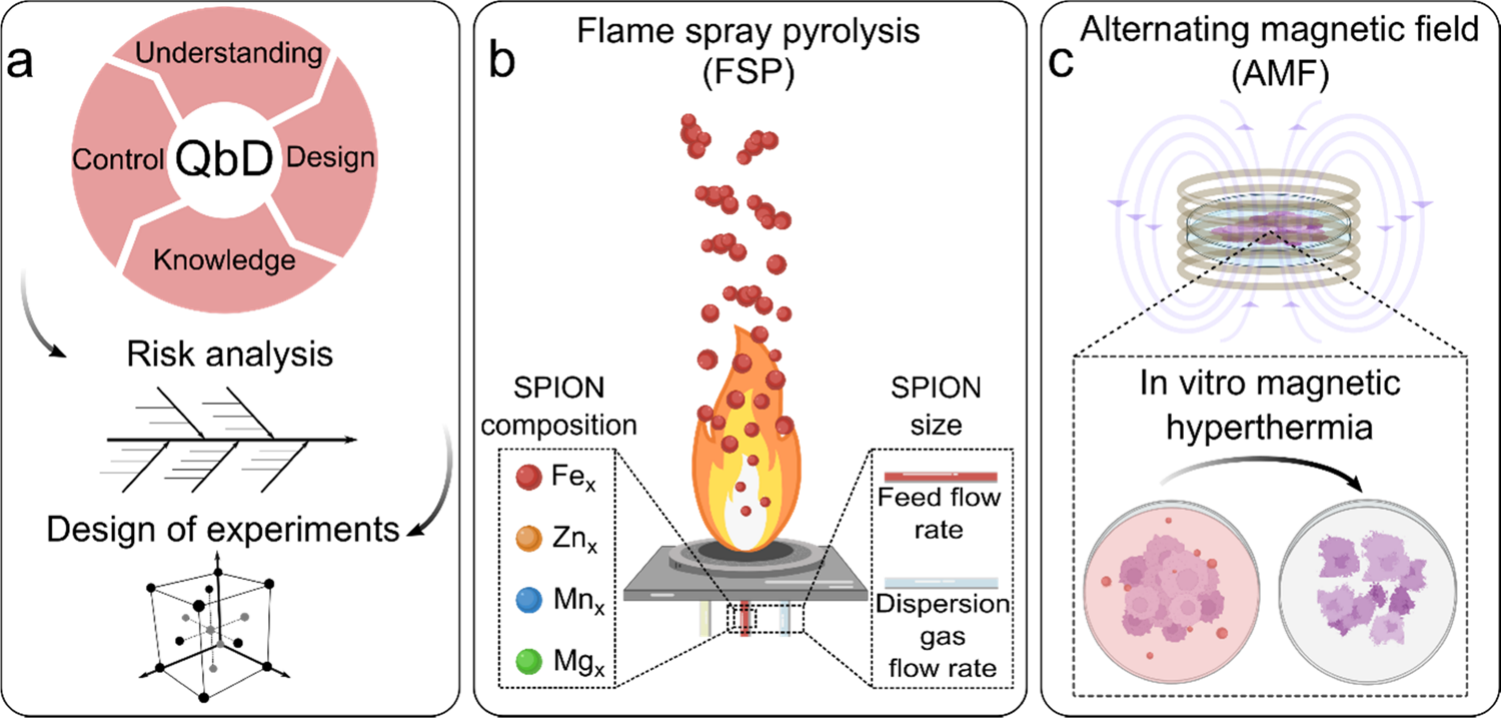
Quality by Design (QbD) Approach for the Development of High-Performance Superparamagnetic Iron Oxide Nanoparticles (SPIONs) for Magnetic Hyperthermia (a) QbD process implementing risk analysis and design of experiments, (b) synthesis of undoped and doped SPIONs by flame spray pyrolysis (FSP), and (c) *in vitro* magnetic hyperthermia using optimized SPIONs.

## Results and Discussion

### Risk Assessment, QTPP, CQAs, and CPPs

**Figure 1:**
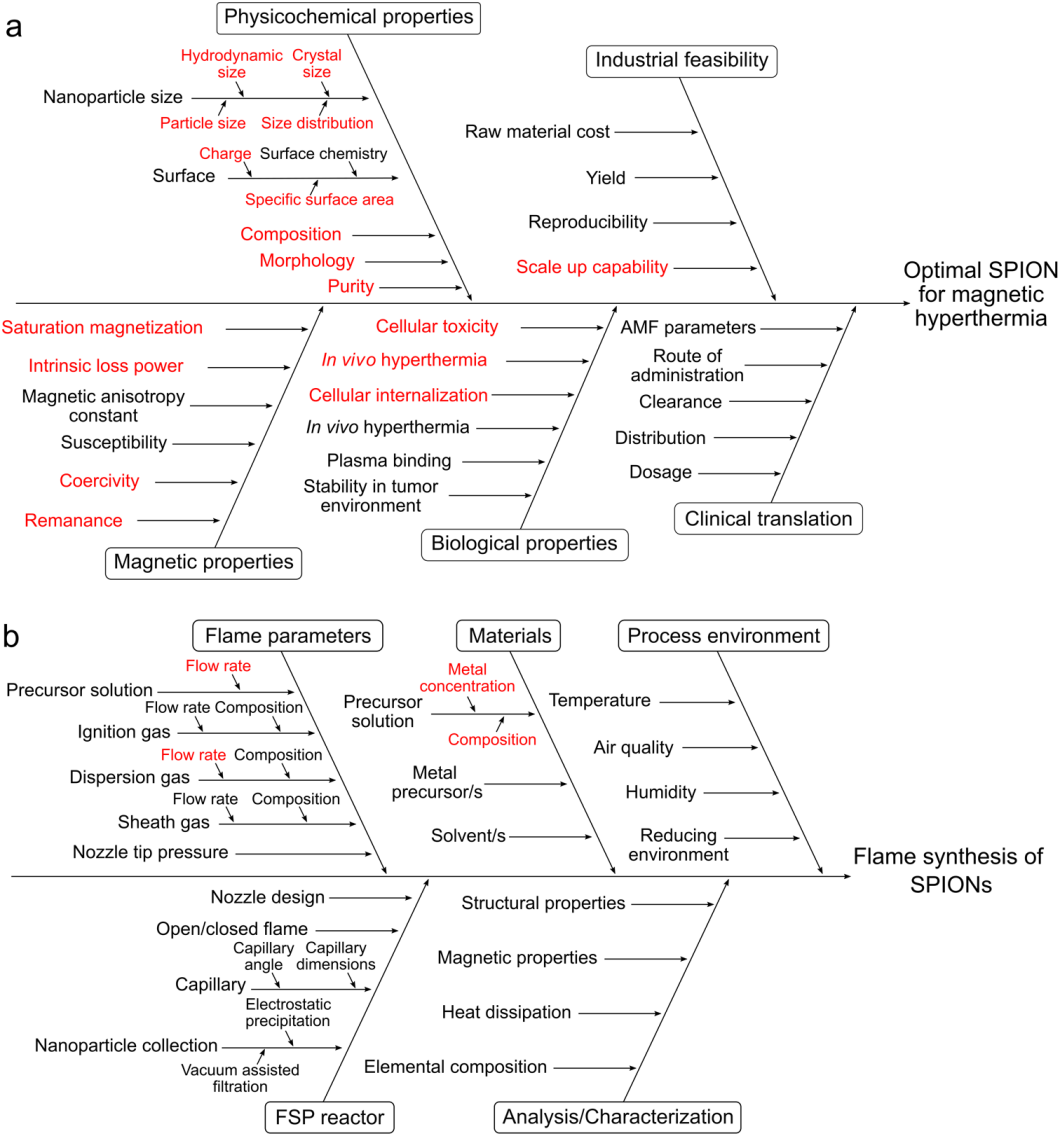
Risk assessment using an Ishikawa diagram to define the critical quality attributes (CQAs) and critical process parameters (CPPs). (a) Quality attributes of an optimal SPION for magnetic hyperthermia and (b) FSP process to produce SPIONs. The CQAs and CPPs evaluated in this study are highlighted in red.

A risk assessment was conducted using an Ishikawa diagram to analyze factors influencing the clinical outcome of magnetic hyperthermia (Figure 1a). This assessment was used to establish QTPP and identify CQAs (Table 1). The QTPP of a magnetic nanoparticle formulation is defined based on the intended therapeutic use. Therefore, the intrinsic loss power (ILP), a measure of heat dissipation from SPIONs, was considered a primary CQA for SPIONs intended for magnetic hyperthermia. A study by Kallumadil et al.^29^ determined the average ILP value for 16 commercially available SPION formulations to be 1.4 nH m^2^ kg^–1^. As a result, we also chose this threshold for the QTPP. Previous studies have shown that maximum heat dissipation tends to occur at the diameter where the ferrimagnetic state of the magnetic domain transitions to a superparamagnetic state.^30^ However, the mechanism of heat loss is very sensitive to the particle size polydispersity, anisotropy constant, aggregation/agglomeration, and particle concentration. Various experimental and computational studies suggest an optimal nanoparticle size for heat dissipation to range from 12 to 20 nm.^31^ This variation is a result of the use of various synthesis techniques, surface coatings, and changes in the magnetic anisotropy constant caused by doping of the SPIONs. In addition, crystal size, rather than primary particle size, has been reported to influence heating efficiency.^22^ Therefore, we identified the average crystal size as a CQA. As saturation magnetization (*M*_s_) and coercivity (*H*_c_) are linked to magnetic hyperthermia and superparamagnetism, respectively, these were also chosen as CQAs.^5^ Composition, specific surface area (SSA), hydrodynamic size, size distribution, and remanence of SPIONs were evaluated as secondary CQAs. Finally, purity, surface charge, particle size, morphology, and cytotoxicity were identified as CQAs and assessed for a subset of key nanoparticles in the DoE. The scale-up capability, *in vitro* hyperthermia, and cellular internalization were demonstrated on the final optimal SPION. Not all of the variables influencing magnetic hyperthermia efficacy were explored within the scope of the current study, but could be addressed in future studies.

**Table 1:** Quality Target Product Profile (QTPP) for SPIONs

critical quality attribute (CQA)	target	justification
intrinsic loss power (ILP) [nH m^2^ kg^–1^]	1.4	hyperthermia performance comparable to commercial SPIONs^29^
crystal size (*d*_XRD_) [nm]	12–20	optimal size for high heat dissipation within a superparamagnetic regime
saturation magnetization (*M*_s_) [emu g_Metal_^–1^]	>65	correlated with hyperthermia performance, with a minimum target based on *M*_s_ of undoped SPIONs^23^
coercivity (*H*_c_) [mT]	<1	ensures superparamagnetism

Industrial feasibility is a critical factor for synthesizing phase-pure inorganic nanoparticles on a large scale. Thus, FSP was chosen as the synthesis method due to its proven scalability, reproducibility, and control over the size and composition of SPIONs.^32,33^ Additionally, FSP is a relatively sustainable manufacturing technique because it uses low-cost precursors, such as nitrates and organic solvents like ethanol.

The Ishikawa diagram of the FSP process identified the variables potentially affecting the CQAs, and consequently magnetic hyperthermia (Figure 1b). The diagram categorizes the variables of flame synthesis of SPIONs into five groups, which are further divided into subgroups. The variables were ranked on severity, occurrence, and detectability using failure mode and effects analysis from previous studies (Table S2).^26^ The precursor flow rate, dispersion gas flow rate, and precursor concentration are known to influence nanoparticle size during flame synthesis,^19,20^ so they were ranked with a high-risk priority number (>15). An initial DoE (Table S3) was constructed to link and quantify the effect of FSP parameters on the size of undoped SPIONs, using a central composite orthogonal fractional factorial design (Figure S1a).

Although precursor solution composition had a moderate risk priority number in the flame synthesis process, it significantly impacts the SPION composition and thus their magnetic and heat dissipation properties. Doping iron oxide nanoparticles with metals such as manganese, zinc, and magnesium drastically affects the structural and magnetic properties and heating performance.^22,34−37^ The relative amount of the dopant also plays a crucial role in producing structural and magnetic changes. For instance, Zn content in the range 0.3 < *x* < 0.5 produces the most efficient heating.^38,39^ Given that SPION composition is a key factor in improving the properties of magnetic nanoparticles for biomedical applications, a second DoE (Table S3) was constructed using a D-optimal design (Figure S1b). This one modeled the impact of average crystal size, choice of dopant metal, and dopant concentration on the heating performance and magnetic properties of SPIONs.

### Physicochemical Characterization of Undoped SPIONs

**Figure 2:**
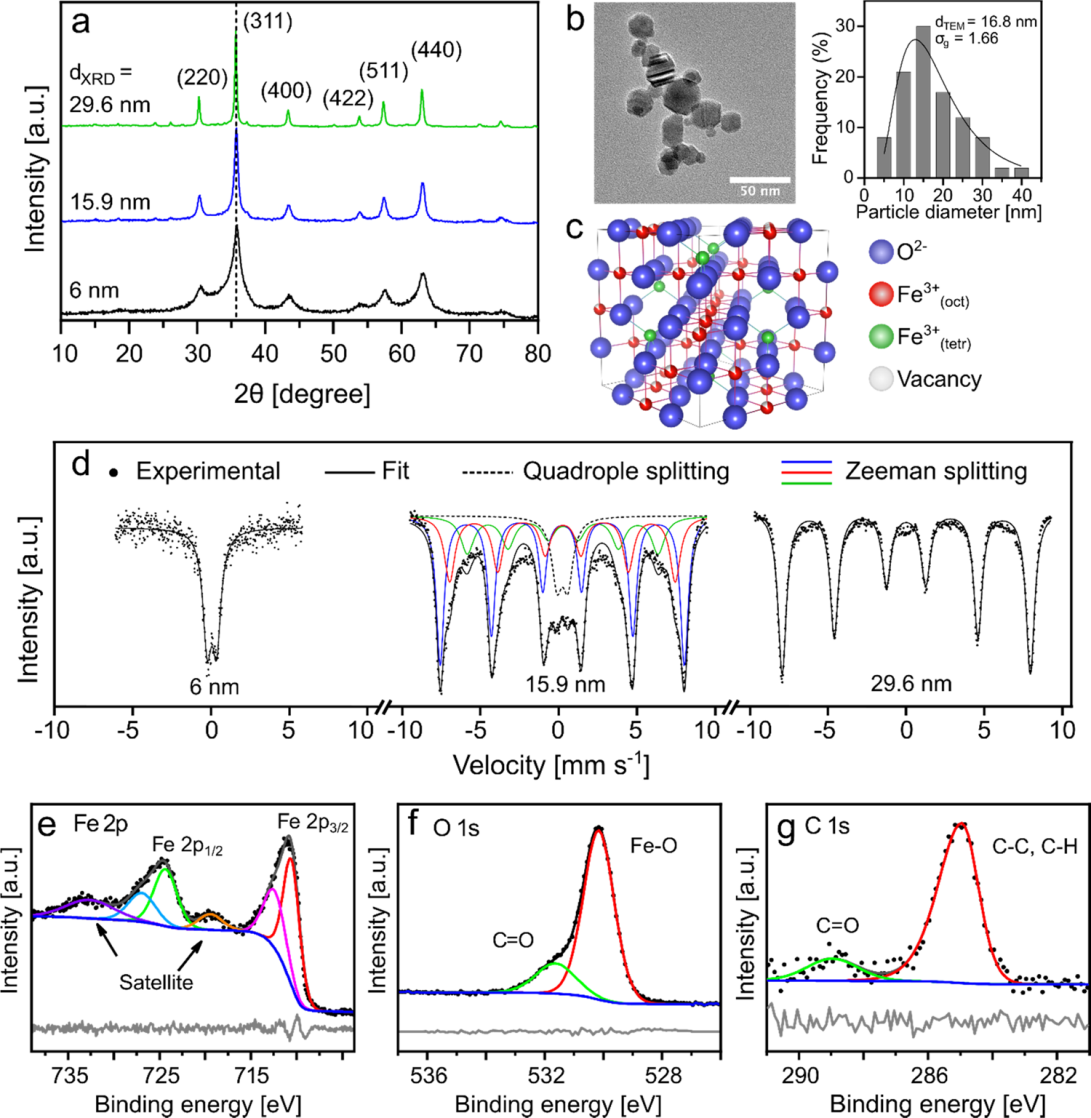
Physicochemical properties of undoped SPIONs. (a) XRD patterns of γ-Fe_2_O_3_ nanoparticles with crystallite (*d*_XRD_) sizes of 6 (black), 15.9 (blue), and 29.6 nm (green). The dashed line represents the maghemite (311) peak. (b) TEM image and corresponding particle size distribution of γ-Fe_2_O_3_ 15.9 nm nanoparticles. (c) Crystal structure of a cubic spinel unit cell of γ-Fe_2_O_3_. (d) Mössbauer spectra of 6, 15.9, and 29.6 nm γ-Fe_2_O_3_ nanoparticles at 295 K. XPS spectra of 15.9 nm γ-Fe_2_O_3_ nanoparticles showing the core levels of (e) Fe 2p, (f) O 1s, and (g) C 1s.

Different sizes of undoped SPIONs were produced based on the initial DoE design (Table S3) to systematically investigate the effect of FSP process parameters on nanoparticle size. Table S4 lists the average crystal sizes (*d*_XRD_) and lattice constants derived from X-ray diffraction (XRD), along with the SSA obtained from the Brunauer–Emmett–Teller method for all particles. Figure 2a shows the XRD patterns for three representative nanoparticle sizes in the low, mid, and high regions of our size range. The diffraction peaks of all samples correspond to cubic spinel structures, showing six prominent peaks originating from the (220), (311), (400), (422), (511), and (440) crystallographic planes.^40^ The 6 nm SPION exhibits a broadened pattern, attributed to the fewer number of reflection planes in smaller particles.^41^

Thermogravimetric analysis (TGA) was performed to determine the purity of the flame-made nanoparticles (Table S4). The sample weight was normalized at 120 °C to exclude the loss of physisorbed water from the nanoparticle surface.^42^ The weight loss from 120–900 °C is attributed to the decomposition of precursor and solvent residues from the nanoparticle surface, thereby releasing NO_*x*_ and CO_*x*_ products.^20,21^ Moreover, the 6 nm γ-Fe_2_O_3_ nanoparticles lost 4.73 wt %, markedly higher than the 15.9 nm (3.57 wt %) and 29.6 nm SPIONs (2.24 wt %). This could be due to the large SSA of small nanoparticles (Table S4), which would lead to a high amount of surface-adsorbed residues. The overall low weight loss in all samples (<5%) indicates the high purity of flame-made SPIONs.

The morphology and particle size of the γ-Fe_2_O_3_ nanoparticles were determined through transmission electron microscopy (TEM). A representative TEM image is shown in Figure 2b of γ-Fe_2_O_3_ nanoparticles with an average crystal size of 15.9 nm. The nanoparticles are polyhedron-shaped, typical of flame-made SPIONs.^23,32,38,43^ A primary particle size (*d*_TEM_) of 16.8 nm was calculated through a log-normal fitting of the particle size distribution. The small (*d*_XRD_ = 6 nm) and large (*d*_XRD_ = 29.6 nm) sized γ-Fe_2_O_3_ nanoparticles showed *d*_TEM_ values of 9.5 and 30.3 nm, respectively (Figure S2). The good agreement of *d*_XRD_ and *d*_TEM_ indicates monocrystalline particles. The SPIONs exhibit a geometric standard deviation (σ_g_) of 1.3–1.7, which is close to the self-preserving size distribution of nanoparticles produced by aerosol synthesis.^40,44^

Iron oxide nanoparticles for hyperthermia applications are primarily synthesized as maghemite (γ-Fe_2_O_3_) or magnetite (Fe_3_O_4_). Both have a cubic spinel crystal structure in which Fe ions are distributed in tetrahedrally coordinated A-sites and octahedrally coordinated B-sites. All unit cell positions are occupied in magnetite, whereas the maghemite crystal structure has vacancies due to incomplete occupation of the B-sites. Figure 2c shows a unit cell of maghemite where the octahedral vacancy is represented by partially red Fe^3+^ ions. For reproducible magnetic properties, it is crucial to establish the phase of the iron oxide nanoparticles. Magnetite has a better heating performance than maghemite,^45^ but XRD cannot reliably distinguish between these two phases due to overlapping peak positions and intensities of the crystal structures. Therefore, Mössbauer spectroscopy was performed to study the phase composition and crystal structure of the iron oxide nanoparticles (Figure 2d). The fitting was performed using an electric quadrupole split doublet for the 6 nm SPIONs, a central doublet and several six-line patterns for the 15.9 nm SPIONs, and a single six-line pattern for the 29.6 nm SPIONs. The results from the fitting are presented in Table S5. Figure 2c shows a quadrupole doublet in the small- and midsized SPION sample. This indicates single domain particles originating from the collapse of a hyperfine field caused by a faster magnetic relaxation rate than the Mössbauer measurement time.^46,47^ The absence of the doublet in the 30 nm SPIONs could be due to the smaller fraction of superparamagnetic particles. A significant fraction of the particles exhibits sizes above the superparamagnetic limit due to the inherent size distribution from the aerosol process (Figure S2b). The isomer shift values were used to identify the phase composition since they do not depend on the particle volume. The observed averaged isomer shifts of 0.32–0.34 mm s^–1^ correspond more closely to that of maghemite (0.32 mm s^–1^) than magnetite (0.51 mm s^–1^).^48^ This strongly indicates that the undoped SPIONs mainly consist of maghemite, which is also in excellent agreement with the literature.^40,41^ The production of phase-pure maghemite through FSP can be attributed to the high-temperature flame and the highly oxidizing environment of the process.

The chemical composition of undoped SPIONs was further investigated by X-ray photoelectron spectroscopy (XPS) of the 15.9 nm nanoparticles. Figure 2e shows the binding energy peaks at 710.8 and 724.6 eV, corresponding to Fe 2p_3/2_ and Fe 2p_1/2_, respectively, which closely match the characteristic binding energy of Fe^3+^ ions.^49,50^ Satellite peaks at 719.4 and 732.9 eV also suggest the presence of Fe^3+^ ions, indicating that the sample primarily consists of a maghemite phase. The O 1s spectrum (Figure 2f) illustrates two peaks at 530.1 and 531.6 eV, which can be assigned to the oxygen bonded to carbon and the lattice oxygen in γ-Fe_2_O_3_, respectively. Figure 2g shows the C 1s spectrum comprising a peak at 288.9 eV (characteristic for carbon bonded to oxygen) and another peak at 284.9 eV. These peaks indicate the presence of adventitious carbon on the nanoparticle surface, usually caused by exposure to air or sample handling during measurement.^49,51,52^ This was additionally indicated by the loss of up to 4.7 wt % in the TGA analysis, which we attributed to the combustion of carbon residues on the nanoparticles (Table S4). Mössbauer spectroscopy and XPS results confirm that the undoped SPIONs are composed of maghemite.

### Modeling of the SPION Crystal Size

**Figure 3:**
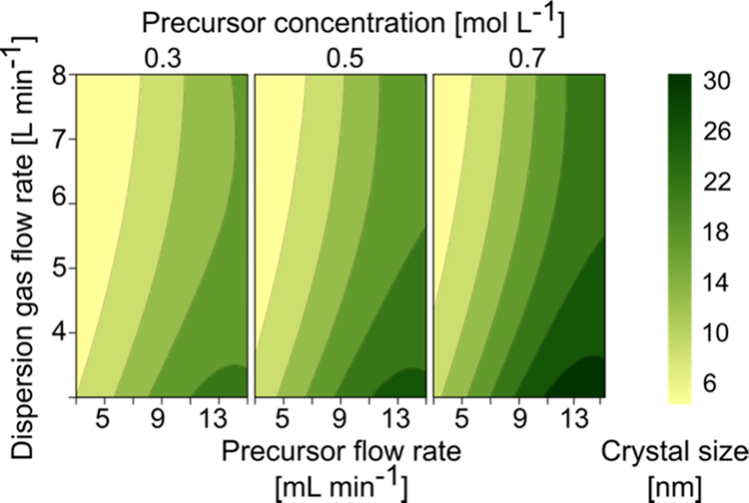
Three-dimensional (3D) contour plot of the crystal size of undoped SPIONs as a function of precursor concentration, flow rate, and dispersion gas flow rate in the FSP reactor. The color scale indicates the average crystal size obtained from Rietveld refinement and the Scherrer equation using XRD.

Multiple linear regression was used to model the average crystal size and SSA of the undoped SPIONs (Table S6). The adjusted model resulted in an excellent fit for crystal size (*R*^2^ = 0.97 and *Q*^2^ = 0.95) and SSA (*R*^2^ = 0.97 and *Q*^2^ = 0.95). A strong correlation was obtained between the predicted and measured values (Figure S3). Figure 3 shows the effect of FSP parameters on crystal size, with the precursor flow rate having the strongest positive influence (Table S6). Average crystal size increased with increasing precursor flow rate and plateaued at very high flow rates, indicating a negative quadratic effect (Figure 3); this could be a result of the experimental design. The use of a low dispersion gas flow rate at high precursor flow rates leads to difficulties in obtaining a fine precursor spray. This may then affect the combustion of the precursor and subsequent particle formation. The dispersion gas flow rate had a negative effect on the crystal size with a significant positive quadratic influence. The precursor concentration also showed a significant influence on the size of undoped SPIONs, attributed to increased flame enthalpy and mass concentration, resulting in increased particle collision and sintering rates.^33,53^ This leads to the formation of larger nanoparticles, which is in excellent agreement with the literature.^19,20,33^

The precursor and dispersion gas flow rates interacted significantly with each other. Particle size increased as the ratio of precursor-to-dispersion gas flow rate increased, which is due to the longer residence time of particles at high temperatures. This extended time promotes particle sintering and coagulation rate, resulting in the increase of the primary particle size.^54^ The resulting predictive model was used to compute the required process parameters to synthesize doped SPIONs of three sizes (6, 15, and 30 nm). The relationship between SSA and process factors demonstrated a strong resemblance to the trends seen for crystal size (Table S6). These results are in excellent agreement with the experimental and theoretical framework established for flame synthesis of nanoparticles. This study established a predictive model for the synthesis of SPIONs using DoE.

### Physicochemical Characterization of Doped SPIONs

The D-optimal design was used to produce doped SPIONs incorporating zinc, manganese, or magnesium at three different target crystal sizes (6, 15, and 30 nm). For clarity, these nanoparticle groups are hereafter referred to as small (S), mid (M), and large (L), respectively. The selection of zinc, manganese, and magnesium as dopants was based on their reported ability to significantly affect the magnetic properties and heating performance of SPIONs.^22,34−37,55,56^ All nanoparticles were produced using the same total metal concentration (0.7 M), while the flow rates of precursor and dispersion gas were changed according to the target crystal size. Inductively coupled plasma optical emission spectroscopy (ICP-OES) confirmed the dopant content in SPIONs, and showed dopant-to-iron ratios consistent with their calculated stoichiometric mass ratios (<10% relative error) (Table S7). Furthermore, TGA analysis revealed that the midsized doped SPIONs exhibited a small weight loss (2–4 wt %; Table S7), likely due to the thermal decomposition of the carbon residues. Trace carbon is not expected to negatively affect biomedical applications, given that studies have explored biocompatible carbon coatings on magnetic nanoparticles.^57,58^ Our findings validate the use of FSP for robust and facile production of doped SPIONs with well-defined particle composition and high purity, crucial to controlling the heating performance of SPIONs.

**Figure 4:**
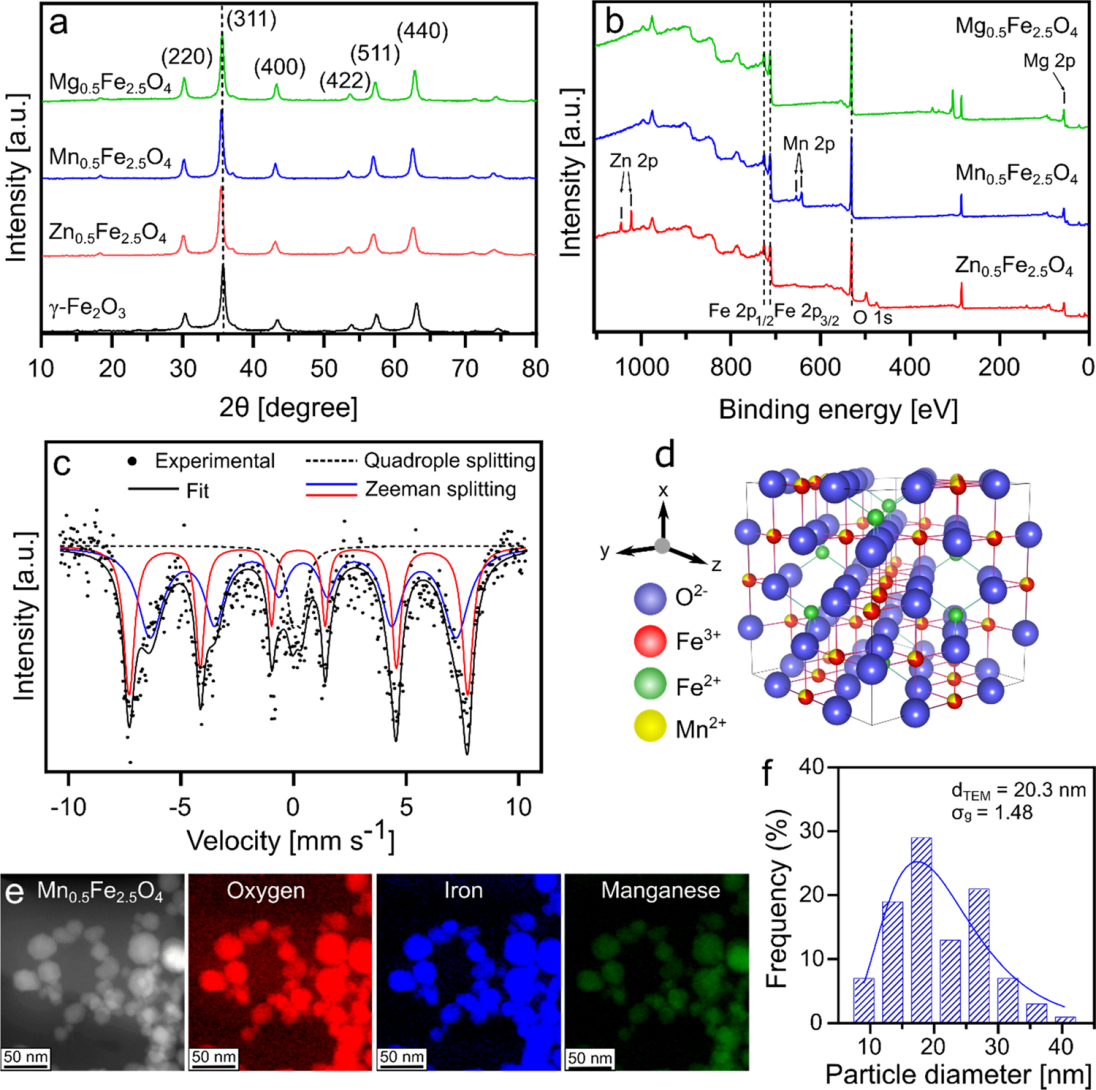
Physicochemical properties of midsized (∼15 nm) doped SPIONs. (a) XRD pattern of γ-Fe_2_O_3_ (black), Zn_0.5_Fe_2.5_O_4_ (red), Mn_0.5_Fe_2.5_O_4_ (blue), and Mg_0.5_Fe_2.5_O_4_ (green). The dashed line represents the maghemite (311) peak. (b) XPS survey spectra of Zn_0.5_Fe_2.5_O_4_ (red), Mn_0.5_Fe_2.5_O_4_ (blue), and Mg_0.5_Fe_2.5_O_4_ (green). (c) Mössbauer spectrum of Mn_0.5_Fe_2.5_O_4_. (d) Crystal structure of a cubic spinel unit cell of Mn_0.5_Fe_2.5_O_4_. (e) TEM image and elemental mapping images with false coloration for element identification of Mn_0.5_Fe_2.5_O_4_ nanoparticles (red: oxygen, green: manganese, blue: iron). (f) Particle size distribution of Mn_0.5_Fe_2.5_O_4_ nanoparticles.

Figure 4a shows the XRD patterns of midsized undoped SPIONs and doped ferrites Zn_0.5_Fe_2.5_O_4_, Mn_0.5_Fe_2.5_O_4_, and Mg_0.5_Fe_2.5_O_4_. The six prominent peaks originating from the (220), (311), (400), (422), (511), and (440) crystallographic planes correspond to a cubic spinel structure,^59,60^ indicating the presence of maghemite or magnetite phase. No peaks corresponding to other iron oxide phases or metal oxides were observed in any of the samples (Figure S4). The crystallite sizes and lattice parameters are shown in Table S7. Zn^2+^ and Mn^2+^ doped SPIONs showed a slight shift in the diffraction peak toward lower angles (indicated in Figure 4a as a dashed line at (311)). The peak shift and the lattice parameters of these SPIONs increased with a higher dopant concentration (Figure S4a–d). This can be attributed to the difference in ionic radii of Fe^3+^ (0.64 Å) compared to Zn^2+^ (0.74 Å) or Mn^2+^ (0.81 Å).^55,61^ However, this peak shift and lattice expansion was not prominent in the Mg^2+^ doped SPIONs (Figure S4e–f), which can be ascribed to the ionic radii of Mg^2+^ (0.65 Å) being almost equal to that of Fe^3+^.^61^ The peak shift, lack of other metal oxide phases, and the lattice expansion collectively indicate the successful incorporation of dopants into the iron oxide crystal structure.^36,61−63^ Under identical FSP process parameters, increasing the dopant concentration of Mn^2+^ and Mg^2+^ in SPIONs produced no appreciable change in their average crystal sizes compared to that in undoped SPIONs. However, increased Zn^2+^ doping considerably reduced the average crystal size of the SPIONs. The reason is not clear from our results, but similar observations have been reported previously.^62,64^ Finally, three identically made SPION batches exhibited identical diffraction patterns (Figure S5), showcasing the high batch-to-batch reproducibility of the FSP.

The high temperature, oxygen-rich flame conditions during the synthesis of SPIONs, typically lead to a completely oxidized iron oxide phase (maghemite). However, the introduction of dopants such as Zn^2+^ favor the formation of magnetite phase, although the precise mechanism behind this phenomenon remains unclear.^22,38^ To evaluate the elemental and phase composition of the nanoparticles, midsized doped ferrites were characterized by XPS. Similar to undoped SPIONs (Figure 2d), doped SPIONs exhibit the characteristic peaks assigned to Fe 2p_3/2_ and Fe 2p_1/2_ (Figure 4b). The deconvolution of these peaks suggests that the crystal lattice is composed of Fe^3+^ and Fe^2+^ (Figure S6a,d,g), indicating a magnetite crystal lattice. Moreover, additional peaks were observed for doped nanoparticles at 1021.7 641.8, and 49.7 eV for Zn^2+^, Mn^2+^, and Mg^2+^, respectively (Figure S6b,e,h). The small peaks observed at 500 and 300 eV for Zn and Mg-doped SPIONs, respectively, were assigned to Auger electrons, which are produced by the surplus energy of the dopant atoms during relaxation.^65^ These findings collectively indicate the successful integration of dopants into the magnetite lattice structure.^51^

Further elucidation of the phase composition and cation distribution in doped SPIONs was performed by using Mössbauer spectroscopy. Figure 4c shows the Mössbauer fitting patterns obtained for midsized Mn_0.5_Fe_2.5_O_4_ nanoparticles, chosen based on previous reports of promising hyperthermia performance for this particle composition.^23^ The fitting was performed by using a central doublet and two sextet patterns. As previously discussed, the presence of doublet patterns indicates superparamagnetism. The two patterns of sextet correspond most likely to the Fe^2+^ and Fe^3+^ at the A- and B-sites in the cubic spinel crystal lattice. Table S4 shows the Mössbauer parameters of the A and B-sites obtained from the fitting. The isomer shifts of 0.33 and 0.55 mm s^–1^ closely matched the magnetite isomer shifts for the A-site (0.27 mm s^–1^) and B-site (0.63 mm s^–1^), respectively. Additionally, the sextet intensity ratio of 1:1.4 ± 0.2 corresponds well with prior studies on Mn-doped magnetite nanoparticles, indicating the preferential occupation of Mn^2+^ at the B-site.^66,67^ This is shown in Figure 4d as an illustration of the crystal structure of magnetite, where the B-sites are occupied by Mn^2+^ and Fe^3+^ ions. It should be noted that the occupancy of the dopant in the A or B-sites is governed by several factors, such as nanoparticle size and choice and concentration of dopant. Zn^2+^ has been reported to preferentially occupy the A-site, but can potentially exhibit inversion of preferential occupation at higher dopant concentration or smaller particle size.^62,68^ Similarly, previous studies on Mg^2+^ doped SPIONs have demonstrated that at *x* ≤ 0.5, the Mg content is entirely associated with the B-site. However, at *x* > 0.5 the Mg^2+^ ions start to populate both A and B-sites.^69,70^

Detailed morphological characterization of Mn_0.5_Fe_2.5_O_4_ was conducted by using scanning TEM combined with energy-dispersive X-ray spectroscopy and energy-loss spectroscopy. Figure 4e illustrates the high-angle annular dark-field scanning TEM images and corresponding elemental maps.^71,72^ The nanoparticles exhibit a polyhedron shape, which is in good agreement with previously reported flame-made SPIONs.^22,23,43^ The elemental mapping indicates a uniform distribution of the dopant throughout the nanoparticle. The energy-dispersive X-ray spectra (Figure S7a) also show the elemental composition of Mn_0.5_Fe_2.5_O_4_ nanoparticles to be in excellent agreement with the ICP-OES results. Log-normal fitting of the particle size distribution yielded a *d*_TEM_ of 20.3 nm, which closely aligns with the *d*_XRD_ (17 nm) of these nanoparticles, in agreement with our prior investigation (Figure 4f).^23^ The geometric standard deviation (σ_g_ = 1.48) also showed excellent agreement with the self-preserving size distribution of flame-made nanoparticles. Figure S7b shows the Bragg Vector Map calculated from a four-dimensional (4D) scanning diffraction acquisition over a large collection of the Mn_0.5_Fe_2.5_O_4_ nanoparticles.^73^ The Bragg vector map reveals a set of rings due to the random orientation of the crystallites, also shown by the real-space crystal orientation map (Figure S7c). The map fits well with a simulated diffraction pattern of Mn_0.5_Fe_2.5_O_4_ (overlay with additional indexing provided for the intense crystallographic planes in Figure S7b). This also supports the formation of the cubic spinel phase of the doped SPIONs in agreement with XPS and Mössbauer analyses.

### Modeling of Magnetic Properties

**Figure 5:**
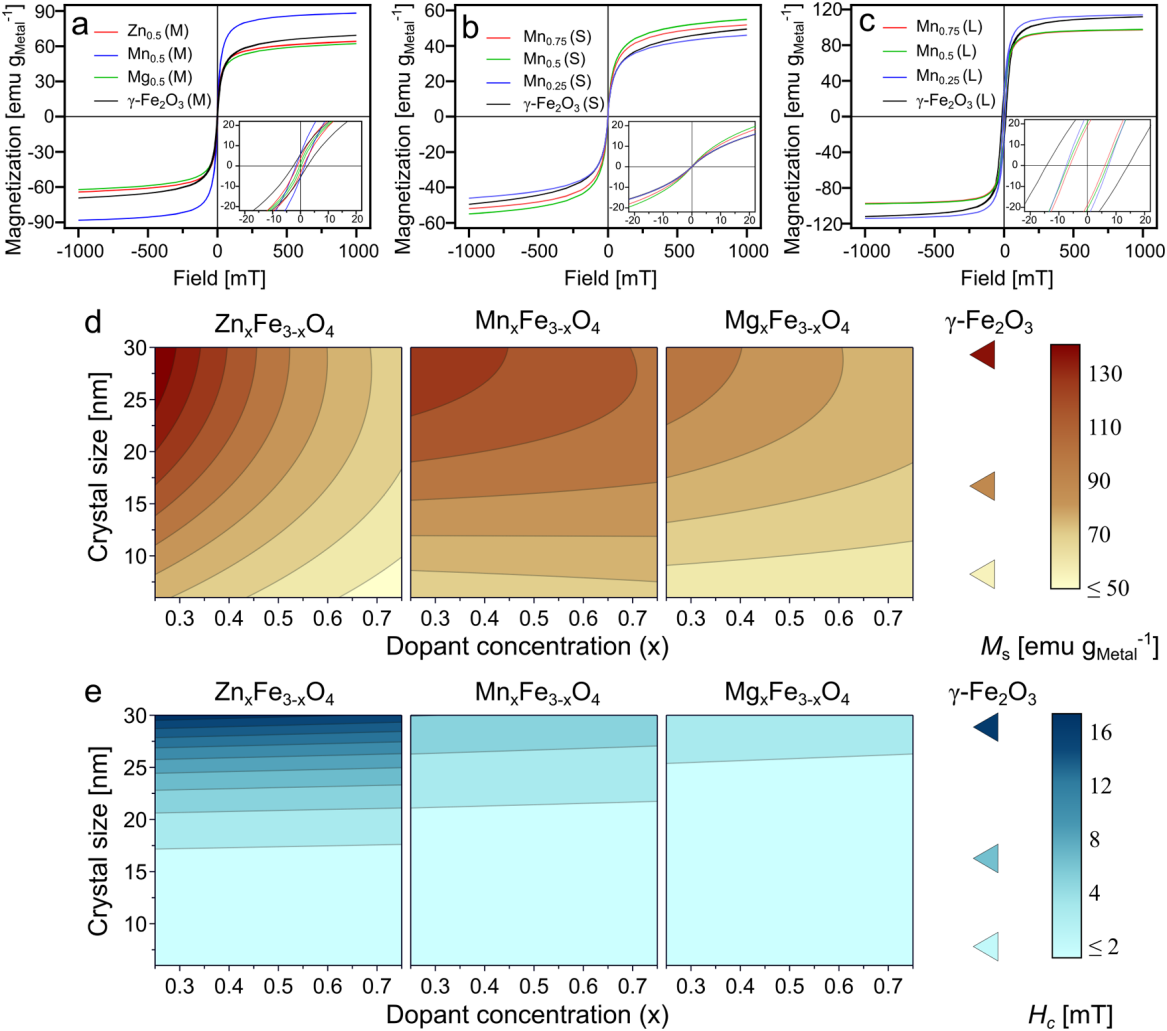
Magnetization curves at 300 K for different particle sizes and compositions. (a) Midsized nanoparticles: γ-Fe_2_O_3_ (black), Zn_0.5_Fe_2.5_O_4_ (red), Mn_0.5_Fe_2.5_O_4_ (blue), and Mg_0.5_Fe_2.5_O_4_ (green); (b) small-sized nanoparticles: γ-Fe_2_O_3_ (black), Mn_0.25_Fe_2.75_O_4_ (blue), Mn_0.5_Fe_2.5_O_4_ (green), and Mn_0.75_Fe_2.25_O_4_ (red); and (c) large-sized nanoparticles γ-Fe_2_O_3_ (black), Mn_0.25_Fe_2.75_O_4_ (blue), Mn_0.5_Fe_2.5_O_4_ (green), and Mn_0.75_Fe_2.25_O_4_ (red). Insets show the magnification of magnetization curves at ±20 mT. Contour plots showing the effect of the dopant metal, dopant concentration, and crystal size on (d) saturation magnetization (*M*_s_) and (e) coercivity (*H*_c_). Triangles represent the respective values of γ-Fe_2_O_3_ at the indicated sizes. The color scales show the values of *M*_s_ and *H*_c_.

The magnetic properties of undoped and doped SPIONs synthesized according to the D-optimal design are summarized in Table S8. Figure 5a shows the magnetization curves of midsized undoped and doped ferrites at a dopant concentration of *x* = 0.5. The magnetization of the nanoparticles is normalized by the total metal content for an adequate comparison between all ferrites. Doped midsized ferrites show negligible hysteresis, confirming their superparamagnetic nature. Incorporating Zn^2+^, Mn^2+^, or Mg^2+^ in SPIONs do not significantly increase the coercivity values of midsized particles. Figure 5b,c show the magnetization curves of small- and large-sized Mn-doped ferrites. The small-sized ones demonstrate negligible hysteresis (Figure 5b), whereas the large ones exhibit notable hysteresis, indicating the presence of blocked nanoparticle magnetic moments, i.e., a ferrimagnetic state (Figure 5c). Similar trends for hysteresis were observed for Zn- and Mg-doped ferrites (Figure S8).

The magnetization of midsized ferrites (Figure 5a) indicates that Mn^2+^ doped SPIONs have a significantly higher saturation magnetization (*M*_s_) (88 emu g_Metal_^–1^) at *x* = 0.5 than the other nanoparticles. Conversely, there is no appreciable change in *M*_s_ of midsized SPIONs doped with Zn^2+^ and Mg^2+^ at *x* = 0.5. *M*_s_ is reported to be strongly affected by the nanoparticle size, which can be clearly observed here (Figure 5b,c). Increasing the size of the Mn-doped SPIONs resulted in a relative increase in *M*_s_. Likewise, a lower Mn^2+^ concentration of large-sized ferrites resulted in higher *M*_s_, consistent with established literature (Figure 5c).^34^ Large-sized Zn-doped ferrites display a markedly elevated *M*_s_ (127 emu g_Metal_^–1^) at low dopant concentrations (Figure S8a), but at high dopant concentration (*x* = 0.75), their *M*_s_ is lower than that of small-sized γ-Fe_2_O_3_ (Figure S8a). This highlights the essential role of controlling the dopant concentration in modulating the magnetic properties of Zn-doped SPIONs. The relationship between SPION size, composition, and magnetic properties is obviously complex and nonlinear. Therefore, the DoE was used to systematically model and quantify these interactions.

To model the magnetic properties of undoped and doped SPIONs, a partial least-squares regression method was used. Table 2 shows the model terms and the corresponding statistics for each response. *R*^2^ quantifies the proportion of response variation explained by the model, indicating the degree to which the regression model effectively captures the inherent patterns within the raw data. *Q*^2^ measures the proportion of response variation predicted through cross-validation, serving as an additional assessment of the model’s predictive accuracy. Model validity is determined by the lack-of-fit test, which compares model error to pure error; a value exceeding 0.25 indicates the absence of lack-of-fit. Finally, reproducibility assesses the variation among replicates relative to the overall variability. Response modeling produced a good fit for *M*_s_ (*R*^2^ = 0.90, *Q*^2^ = 0.68). Notably, the nanoparticle size showed the strongest positive influence on *M*_s_ (Table 2).

**Table 2:** Adjusted Quadratic Models Showing the Estimated Coefficients and Confidence Intervals (95%) for Saturation Magnetization, Coercivity, and Remanence^a^

	saturation magnetization		coercivity		remanence	
model terms	coefficient	CI (±)	coefficient	CI (±)	coefficient	CI (±)
Zn_*x*_Fe_3–*x*_O_4_	–0.010	0.052	0.202	0.110	0.081	0.076
Mn_*x*_Fe_3–*x*_O_4_	0.054	0.034	–0.027	0.093	0.120	0.071
Mg_*x*_Fe_3–*x*_O_4_	–0.045	0.032	–0.174	0.094	0.201	0.072
dopant concentration (*x*)	–0.052	0.032	–0.015	0.081	–0.098	0.063
crystal size	**0.118**^b^	0.028	**0.804**^b^	0.088	**1.041**^b^	0.070
crystal size * crystal size	–0.054	0.032	–0.129	0.165	–0.446	0.105s
Zn_*x*_Fe_3–*x*_O_4_ * *x*	–0.056	0.047				
Mn_*x*_Fe_3–*x*_O_4_ * *x*	0.036	0.033				
Mg_*x*_Fe_3–*x*_O_4_ * *x*	0.019	0.033				
Zn_*x*_Fe_3–*x*_O_4_ * crystal size			0.137	0.143		
Mn_*x*_Fe_3–*x*_O_4_ * crystal size			0.027	0.101		
Mg_*x*_Fe_3–*x*_O_4_ * crystal size			–0.110	0.102		
*x* * crystal size	–0.022	0.026				
*R*^2^	0.90		0.97		0.99	
*Q*^2^	0.68		0.92		0.96	
validity	0.63		0.72		0.65	
reproducibility	0.99		0.99		0.99	

a The models were derived using partial least-squares regression. All responses were log-transformed.

b **Bold**: factors with the most influence on the given response. CI: confidence intervals.

The predictive model in Figure 5d shows 3D contour plots of *M*_s_ as a function of the crystal size and nanoparticle composition. Large-sized nanoparticles generally exhibited higher *M*_s_ than the smaller ones, with values exceeding that of bulk magnetite (92 emu g_Fe_^–1^).^74,75^ The reduced magnetization in small particles can be attributed to the increase in thickness and mass fraction of the surface spin-disordered layer.^76^ The presence of a negative quadratic effect of size on *M*_s_ denotes a nonlinear relationship, suggesting that *M*_s_ saturates as the crystal size increases.

As previously discussed, the dopants can occupy the tetrahedral and octahedral sites in the crystal lattice of SPIONs. This can influence the magnetocrystalline anisotropy and the magnetic moment of the unit cell, thereby affecting the saturation magnetization.^77^ In general, *M*_s_ was positively affected by the incorporation of manganese and zinc in SPIONs, with manganese exhibiting a more pronounced effect. In contrast, doping with magnesium had a negative effect on *M*_s_. Figure 5d shows that a low dopant concentration (*x* = 0.25) positively influenced *M*_s_, whereas a high dopant concentration (*x* = 0.75) produced a negative effect. This is further demonstrated by the lower values of model interaction terms of Zn or Mg and their concentration (Table 2), compared to those of Mn and its concentration. This can be attributed to the varying occupation of dopant ions in the A- and B-sites of spinel SPION crystal and its effect on the magnetic moment of the particle. The total magnetic moment of a unit cell can be defined as, μ = μ_B-site_ – μ_A-site_. The Zn^2+^ (0 μ_B_) and Mg^2+^ (0 μ_B_) ions at low doping levels (*x* < 0.5) are known to preferentially occupy the A-site by replacing the Fe^3+^ (5.92 μ_B_) ions.^62,69^ This may result in a reduction in the magnetic moment at the A-site and a consequent increase of the magnetic moment of the total unit cell, causing an increase in *M*_s_.^36,78^ However, as the concentration of Zn^2+^ and Mg^2+^ increases, the magnetic moment at the A-site lowers substantially, suggesting a weakening of the antiferromagnetic A–O–B superexchange interactions. This may reduce the total overall magnetic moment, leading to a decrease in the *M*_s_.

Figure 5d illustrates that the concentration of Mn^2+^ had a weaker influence on *M*_s_ compared with the other dopants. This can be attributed to the magnetic moment of Mn^2+^ (5.92 μ_B_), which is similar to that of the Fe^3+^ ions. Consequently, the substitution of Fe^2+^ with a high concentration of Mn^2+^ has a less pronounced effect on the magnetic moment compared to substitution with Zn^2+^ or Mg^2+^.^34^ Mn^3+^ (4.98 μ_B_) has been reported to occupy the octahedral site in manganese ferrite nanoparticles; this could negatively affect the total magnetic moment of the unit cell, and thus decrease *M*_s_.^55^ Our study derived a negative interaction term between dopant concentration and nanoparticle size, suggesting that the effect of dopant concentration on *M*_s_ decreases with an increase in nanoparticle size. This could be the result of the large nanoparticle fraction in the samples reaching values of bulk magnetization.

Modeling of coercivity resulted in an excellent fit (*R*^2^ = 0.97, *Q*^2^ = 0.92), which was used to generate a 3D contour plot (Figure 5e). Coercivity was most strongly influenced by the nanoparticle size. The low coercivity in smaller nanoparticles can be ascribed to their superparamagnetic nature. As the particle size increases, the fraction of large ferrimagnetic nanoparticles increases within the particle size distribution, resulting in higher coercivity of the overall sample. Furthermore, doping with zinc had a stronger positive influence on coercivity compared to manganese and magnesium. The large-sized Mn- and Mg-doped SPIONs showed lower coercivity than undoped SPIONs. It has been reported that the incorporation of Mn^2+^ ions into the SPION crystal lattice decreases coercivity by decreasing the magnetic anisotropy.^79^ Modeling of remanence also produced an excellent fit (*R*^2^ = 0.99, *Q*^2^ = 0.96), showing similar trends as observed for coercivity (Table 2).

### Modeling of Magnetic Hyperthermia

In the biological application of magnetic hyperthermia, it is crucial to maintain the biocompatibility and colloidal stability of the nanoparticles while ensuring optimal heating performance. Therefore, a simple biorelevant citrate coating strategy was adopted prior to the measurement of the magnetic hyperthermia performance of the SPIONs. This approach ensured the uniformity of surface coating across all particles produced in this study and facilitated the formation of stable aqueous suspensions. The presence of citrate coating was confirmed by Fourier transform infrared spectroscopy (FTIR) (Figure S9a,b). The citrate-coated nanoparticles exhibited vibrations corresponding to an Fe–O bond at 555 cm^–1^, confirming the presence of the iron oxide phase. An indication of the citrate coating came from peaks observed at 1625 and 1420 cm^–1^, attributed to the stretching modes of carboxyl groups. The hydrodynamic sizes of citrate-coated SPIONs show large variability (80–3000 nm) (Table S8). This is mainly due to the different core sizes and compositions of the SPIONs investigated in this study; these parameters greatly affect the surface area and chemistry of the particles. The decrease in ζ-potential of the small- and midsized SPIONs was more drastic than that of the large-sized ones, which testifies to the poor stability and large hydrodynamic size of the latter (Table S8). The lower SSA of large particles compared to small ones limits their available surface area for interactions with citrate.

**Figure 6:**
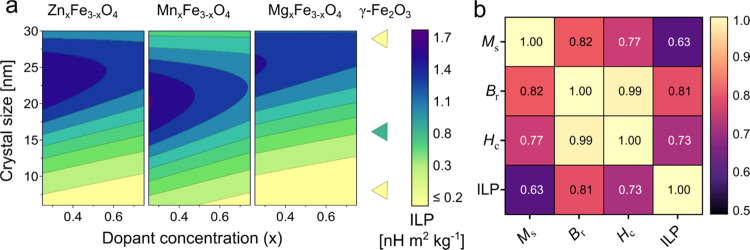
Modeling of intrinsic loss power (ILP) and its correlation with magnetic properties of SPIONs. (a) Contour plots of ILP as a function of crystal size, dopant metal, and dopant concentration. The color scale indicates the ILP. (b) Correlation heatmap reporting the correlation between saturation magnetization (*M*_s_), remanence (*B*_r_), coercivity (*H*_c_), and ILP. The color scale indicates the Pearson correlation coefficients.

The heating efficiency of citrate-coated SPIONs exposed to an AMF was evaluated by using the ILP of these nanoparticles. The ILP measurements were performed in water to ensure biorelevance. Modeling of ILP was performed by partial least-squares regression (Table S9), resulting in an adequate fit (*R*^2^ = 0.87, *Q*^2^ = 0.67). Figure 6a shows the effect of crystal size and the type and concentration of dopant on the ILP of the nanoparticles. The crystal size strongly affected the hyperthermia performance. As crystal size increased, the ILP exhibited an initial rise before peaking and subsequently declined with further increase in size. Previous studies have shown a similar relationship between hyperthermia performance and crystal size of SPIONs at clinical AMF frequencies and amplitudes.^13,80,81^ The heat released from SPIONs is primarily the result of relaxation losses, which can be due to relaxation of the magnetic moment within the particle (Néel relaxation) or rotation of the particle itself (Brownian relaxation). The negative effect of size on nanoparticle heating can be attributed to the exponential growth of the Néel relaxation time with the increase in size. This prolonged relaxation time reaches such high values that it effectively nullifies the relaxation effect at the frequency used for ILP measurements.^82^ In our model, the ILP reached a maximum value between 18 and 25 nm for various nanoparticles, depending on their composition. For monodisperse samples, an optimum particle size of 12–18 nm has been reported to yield the highest heating rate.^80,83,84^ SPIONs with high magnetic anisotropy show low optimal crystal size for magnetic hyperthermia, and vice versa.^85,86^ This can be explained by the exponential correlation of Néel relaxation to *K* × *V*, where *K* is the anisotropy constant and *V* is the crystal volume. Therefore, the variability in the optimal size for heating caused by doping can be attributed to the change in magnetic anisotropy of the nanoparticles.^31,86^

Manganese doping showed the strongest positive effect on ILP, with the highest ILP value (1.9 nH m^2^ kg^–1^) being for midsized Mn_0.5_Fe_2.5_O_4_ nanoparticles. The ILP of these nanoparticles is comparable to the reported hyperthermia performance of commercially available SPIONs such as nanomag-D, Resovist, and fluidmag-D.^29^ Magnesium doping had a negative influence on the ILP indicating a poorer heating performance compared to undoped SPIONs. However, it is important to note that these Mg-doped ferrites aggregated in water, which could have diminished heating abilities in the dispersing medium. The dopant concentrations showed a negative effect on the ILP, exhibiting strong interactions with zinc and magnesium dopants. This indicates that the effect of the change in dopant concentration is more pronounced in zinc and magnesium ferrites than in SPIONs doped with manganese.

The variety of nanoparticles in terms of size and composition posed an additional challenge in achieving sufficiently stable suspensions for hyperthermia measurements. Therefore, the ILP of nanoparticles was also measured in dimethyl sulfoxide (DMSO) by using uncoated nanoparticles. However, modeling of this data resulted in a poor fit (Table S9). The use of the same suspension medium or a uniform coating approach may not suit all particles, leading to dissimilar particle aggregation among different samples. Future studies could focus on tailoring the coating strategy, taking into account the size and composition of nanoparticles, to leverage the systematic approach discussed here.

Figure 6b shows the correlation plot of the measured responses of the nanoparticles. The ILP exhibited a greater positive correlation to the coercivity and remanence of the magnetic nanoparticles than to the saturation magnetization, indicating that magnetocrystalline anisotropy provides the dominating contribution to the magnetic anisotropy. A previous study has reported an increase in hyperthermia performance of zinc-doped SPIONs with a decrease in their coercivity and attributed it to the decrease in interparticle interactions.^22^ Conversely, another study on cobalt-doped SPIONs showed an increase in hyperthermia with an increase in coercivity and attributed it to the increase in magnetic anisotropy combined with the high coercivity.^87^ Our study indicated that an increase in the coercivity and remanence coupled with high *M*_s_, increases the ILP of the SPIONs. High coercivity and remanence are correlated with high magnetic anisotropy,^88^ while a high saturation magnetization denotes the transition from superparamagnetic to blocked regime.^22^ Our study systematically quantifies this relationship between material characteristics and a wide variety of material compositions and sizes.

### Modeling of an Optimal Operating Space and Investigation of *In Vitro* Magnetic Hyperthermia

**Figure 7:**
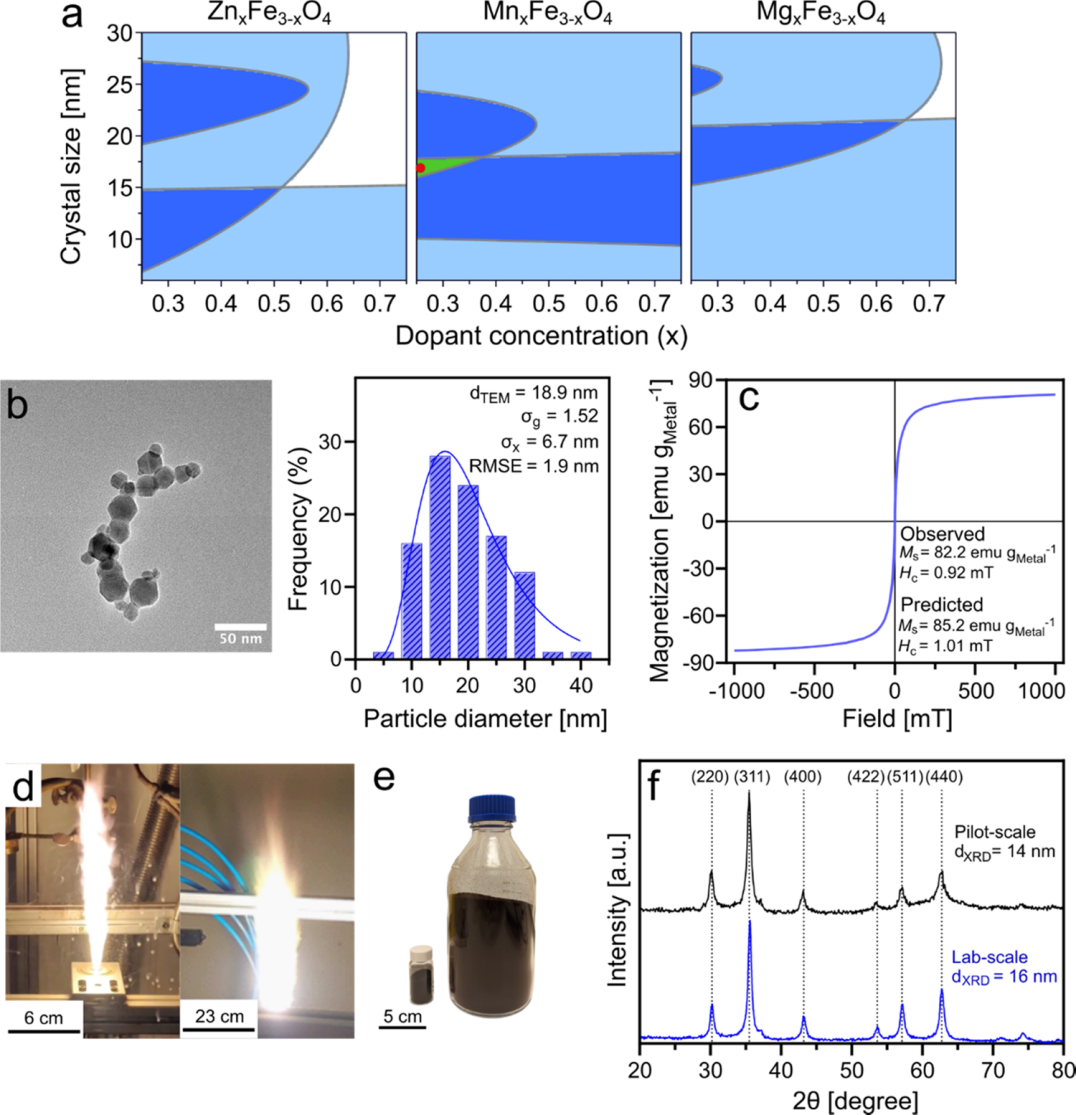
(a) Contour plot modeling of the optimal operating space. Restrictions were set for coercivity (<1 mT), saturation magnetization (>65 emu g_Metal_^–1^), and ILP (>1.4 nH m^2^ kg^–1^). Color code: blue (one or two restrictions met) and green (all restrictions met). The red circle represents the attributes selected for the optimal nanoparticles. (b) TEM image and corresponding particle size distribution of midsized Mn_0.25_F_2.75_O_4_ nanoparticles. (σ_*x*_, arithmetic standard deviation; RMSE, root mean squared error). (c) Magnetization vs magnetic field curves and corresponding magnetic properties of midsized Mn_0.25_F_2.75_O_4_ nanoparticles. (d) Flames of a laboratory-scale FSP reactor (left panel) and a pilot-scale FSP reactor (right panel). (e) Product Mn_0.25_F_2.75_O_4_ powder from a laboratory-scale FSP reactor (left) and a pilot-scale FSP reactor (right). (f) XRD patterns of laboratory- and pilot-scale nanoparticle batches.

The optimal SPION for magnetic hyperthermia was defined based on the QTPP (Table S2). Therefore, a design region was constructed by overlaying the contour plots for all CQAs and applying restrictions for each attribute to comply with the acceptance limits (Figure 7a). Restrictions were set as follows: (i) coercivity (<1 mT), to ensure superparamagnetism and adequate heating; (ii) saturation magnetization (>65 emu g_Metal_^–1^), to maximize the heat loss;^34^ and (iii) ILP (>1.4 nH m^2^ kg^–1^), to ensure a hyperthermia performance comparable to commercial SPIONs.^29^ This identified the optimal operating space meeting all criteria (Figure 7a, green area). This region constitutes a midsized crystal (15–18 nm) and a low-to-medium dopant concentration (0.25 ≤ *x* ≤ 0.5) of Mn. On the basis of the CQAs, the midsized ferrite with a composition of Mn_0.25_Fe_2.75_O_4_ was identified as the optimal nanoparticle for magnetic hyperthermia and synthesized for further characterization. Figure 7b presents the TEM image and corresponding particle size distribution of the FSP-made midsized Mn_0.25_Fe_2.75_O_4_ nanoparticles, exhibiting a *d*_TEM_ of 19 nm and a σ_g_ of 1.52. The magnetic properties of the nanoparticles closely aligned with their predicted values (Figure 7c), with less than 5% relative standard deviation. Citrate coating of the midsized Mn_0.25_F_2.75_O_4_ nanoparticles resulted in a hydrodynamic diameter of 112.9 nm and ζ-potential of −25.6 nm. Notably, their ILP (2.02 nH m^2^ kg^–1^) is higher than that of all the nanoparticles evaluated within the DoE study, and more than 40% higher than the mean ILP of commercial SPIONs.^29^ These findings highlight the strength of the DoE in enabling the discovery of high-performance magnetic nanoparticles.

To demonstrate the scalability of SPION production by FSP, a pilot-scale FSP setup was used to manufacture 100 g of dry Mn_0.25_Fe_2.75_O_4_ nanoparticles at a production rate of 180 g h^–1^ (Figure 7d). This is a 9-fold increase in production rate from the 20 g h^–1^ achieved at the laboratory scale (Figure 7e). The XRD patterns of nanoparticles synthesized at laboratory and pilot scales were identical: both exhibited the characteristic magnetite/maghemite cubic spinel lattice with a crystal size of 14 nm for the pilot-scale batch (Figure 7f). The suitability of large-scale production of γ-Fe_2_O_3_ by FSP has been demonstrated by Estévez et al.^21^ Previous studies on scaling up of FSP have shown that synthesis of phase-pure crystalline nanoparticles can be readily increased by up to 50 times, from production rates of 2–10 g h^–1^, by maintaining a constant gas-to-liquid mass ratio.^18,54^ This facile and successful scale-up of SPION production affirms the feasibility of large-scale manufacturing of high-performance nanoparticles by FSP.

**Figure 8:**
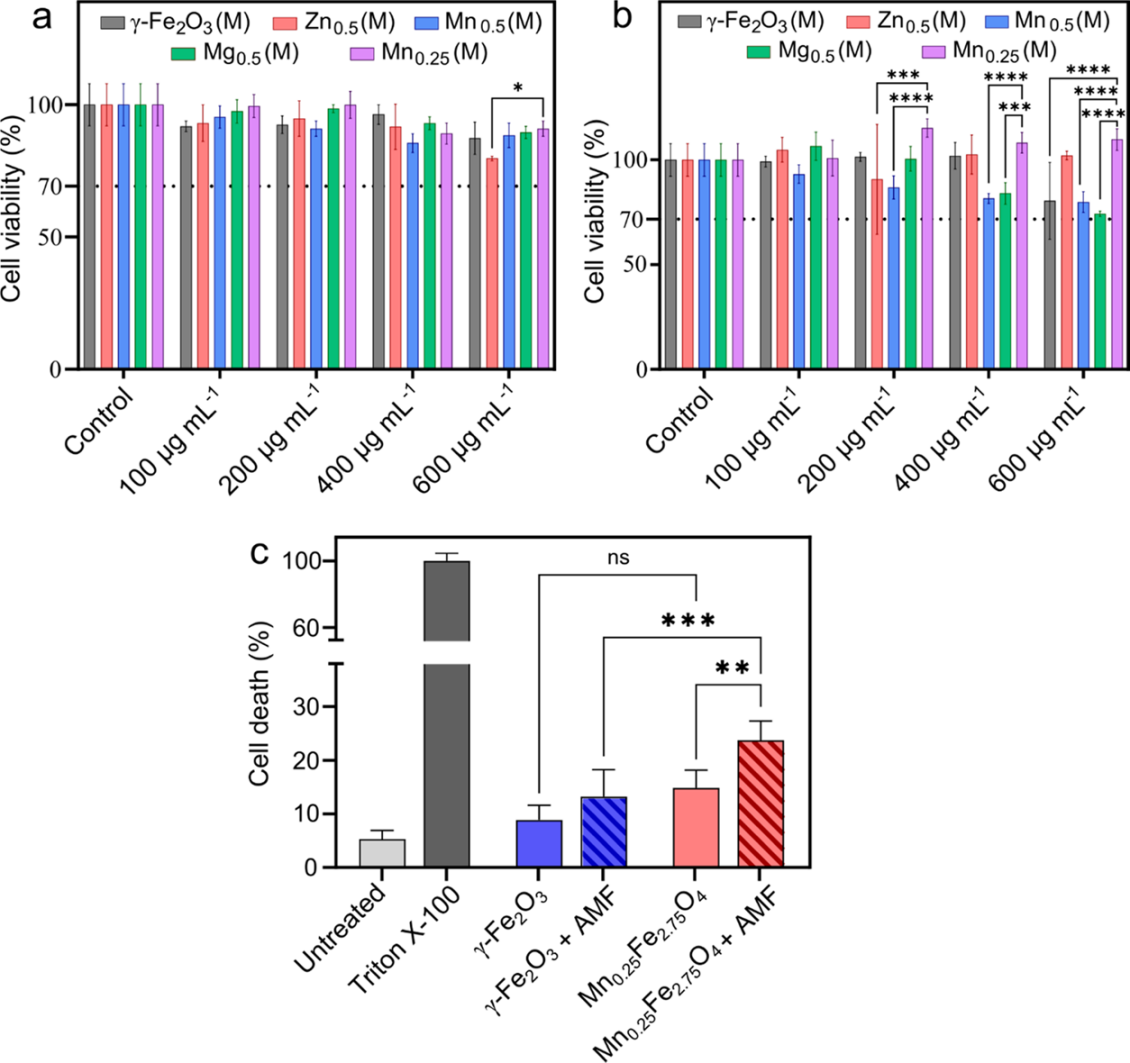
Cell viability of nondifferentiated (a) Caco-2 and (b) HEK-293 cell lines after exposure to midsized γ-Fe_2_O_3_, Zn_0.5_Fe_2.5_O_4_, Mn_0.5_Fe_2.5_O_4_, Mg_0.5_Fe_2.5_O_4_, and Mn_0.25_Fe_2.75_O_4_ nanoparticles at different concentrations (100, 200, 400, and 600 μg mL^–1^). Cell viability was determined using the CellTiter-Glo luminescent cell viability assay and calculated as a percentage of the control. Data show the average of at least four experiments ± standard deviation (SD). (c) *In vitro* hyperthermia performance of midsized Mn_0.25_Fe_2.75_O_4_ (400 μg mL^–1^) nanoparticles against Caco-2 cells in an AMF (14 mT and 592 kHz). *p* < 0.1234 (ns), 0.0332 (*), 0.0021 (**), 0.0002 (***), and 0.0001 (****).

An important aspect of optimal nanoparticle therapy is to minimize associated cytotoxicity by careful dopant selection and low dopant concentration.^23^ We assessed the Mn_0.25_Fe_2.75_O_4_ nanoparticles and other doped ferrites for toxicity using colorectal adenocarcinoma cell lines (Caco-2, SW-480, and HT-29 cell lines) and human embryonic kidney cell line (HEK-293). These cell lines are valuable models for investigating the cytotoxic effects of nanoparticles across both diseased and healthy human cells. Upon observation in an optical microscope, the cells exhibited no signs of unusual distress following incubation with nanoparticles. Figure 8a,b shows the viability of the Caco-2 and HEK-293 cell lines with SPIONs at different concentrations. All nanoparticles exhibited a cell viability exceeding 70%, which surpasses the threshold outlined in the international standard ISO 10993-5 for nontoxicity.^89^ In the Caco-2 cells, Mn_0.25_Fe_2.75_O_4_ nanoparticles exhibited cytotoxicity comparable to that of the other nanoparticles across varying doses, except for Zn_0.5_Fe_2.5_O_4_, which displayed significantly higher cell death than Mn_0.25_Fe_2.75_O_4_ at 600 μg mL^–1^ (Figure 8a). The high cytocompatibility of Mn_0.25_Fe_2.75_O_4_ nanoparticles was more evident for HEK-293 cells, showing significantly superior cell viability across the entire concentration range compared to undoped and doped ferrites (Figure 8b).

The high biocompatibility of Mn_0.25_Fe_2.75_O_4_ was also demonstrated in the SW-480 and HT-29 cell lines (Figure S10). These findings are consistent with prior studies showing a superior toxicity profile of Mn ferrites compared to Zn ferrites.^23,90^ Several *in vivo* studies in rats and mice have shown that pure Mn intravenous doses of up to 5 mg kg^–1^ are well tolerated, with no signs of cardiovascular or neurological disorders.^91^ At a dosage of 1.9 g of nanoparticles per treatment using Mn_0.25_Fe_2.75_O_4_ nanoparticles designed in this study, the formulation administers 169 mg of Mn within the iron oxide crystal. This is equivalent to a dose of 2.5 mg kg^–1^ for a 70 kg individual, which is much below the toxicity threshold determined in preclinical studies. Therefore, based on the magnetic properties, heating performance, and toxicity profile, the midsized ferrite with a composition of Mn_0.25_Fe_2.75_O_4_ was identified as the optimal nanoparticle for magnetic hyperthermia and was further evaluated for its effectiveness in *in vitro* hyperthermia.

The midsized Mn_0.25_Fe_2.75_O_4_ nanoparticles optimized using the QbD approach were evaluated for magnetic hyperthermia against the Caco-2 cells (Figure 8c). The selection of this cellular model aligns with the application of SPIONs in cancer treatment, thus, facilitating a targeted and relevant evaluation. Caco-2 is an adherent and resilient cell line with a high proliferative potential. These characteristics result in underestimation of cell death caused by SPION treatment. Nonetheless, Caco-2 cells are routinely used in preclinical drug development and are an effective model in guiding *in vivo* studies.^92^ The AMF treatment was performed at 14 mT and 592 kHz (*H* × *f* = 6.59 × 10^9^ A m^–1^ s^–1^) for 30 min; these values were selected based on recent comprehensive studies on clinically permissible AMF parameters.^93−97^ Although the conventional Atkinson–Brezovich limit suggests *H* × *f* ≤ 4.85 × 10^8^ A m^–1^ s^–1^ as the acceptable threshold, more recent studies have reported much higher acceptable limits, ranging from 1.8 × 10^9^ to 18.7 × 10^9^ A m^–1^ s^–1^.^93−96^Figure 8c shows the cell death induced by magnetic hyperthermia using citrate-coated midsized Mn_0.25_Fe_2.75_O_4_ nanoparticles. The Mn_0.25_Fe_2.75_O_4_ nanoparticles resulted in 25% mean cell death, which was 80% higher than that induced by midsized γ-Fe_2_O_3_. This indicates a greater cellular heating efficiency of Mn_0.25_Fe_2.75_O_4_ nanoparticles compared to that of undoped γ-Fe_2_O_3_. No significant difference in cell death was observed between the two nanoparticle treatments in the absence of AMF exposure, indicating a similar cellular compatibility of both nanoparticles.

Cellular uptake conducted under conditions identical to cellular hyperthermia revealed that only 1.9 ± 0.1 wt % of Mn_0.25_Fe_2.75_O_4_ and 0.9 ± 0.2 wt % of γ-Fe_2_O_3_ nanoparticles were internalized by the cells. The low cellular uptake indicates that a majority of the nanoparticle fraction remains in the cell medium and the effect of hyperthermia may primarily arise from heat-induced damage to the cell surface. The short incubation time (2 h) and the negatively charged surface coating may contribute to the low internalization of SPIONs in the cells observed here.^98,99^ The impact of different nanoparticle uptake levels and the subsequent intracellular transport on magnetic hyperthermia should be explored in future studies. These investigations should consider the factors influencing cellular internalization such as SPION concentration, incubation time, specific cell line relevant to the target application, and composition of the cell culture media.

## Conclusions

In this study, we report the implementation of a pharmaceutical QbD approach for the development of SPIONs for magnetic hyperthermia. Undoped and doped SPIONs of several sizes and compositions were successfully produced with FSP. Risk assessment and DoE linked the nanoparticle composition and size to their magnetic and heating properties. Hyperthermia performance was strongly influenced by crystal size and SPION composition in complex nonlinear relationships. Moreover, ILP showed a stronger correlation to the coercivity and remanence of the SPIONs than to saturation magnetization. The modeling of CQAs through the optimal operating space identified midsized Mn_0.25_Fe_2.75_O_4_, that fulfilled the QTPP criteria for saturation magnetization, coercivity, and ILP. These nanoparticles were then produced on a pilot scale at a production rate of 180 g h^–1^, affirming the feasibility of large-scale manufacturing. Cytotoxicity investigations across multiple cell lines established the superior cytocompatibility profile of Mn_0.25_Fe_2.75_O_4_ nanoparticles compared to other midsized ferrites. *In vitro* hyperthermia assessment revealed that these nanoparticles induced 80% higher cell death than the γ-Fe_2_O_3_ ones, thereby substantially improving the hyperthermia performance of SPIONs by the use of the QbD approach. Further *in vivo* studies could assess the broader implications of these findings.

The use of FSP to synthesize doped SPIONs is a promising tool for the large-scale and reproducible production of tailored nanoparticles for biomedical applications. Our study demonstrates the advantages of implementing a systematic approach for engineering magnetic nanoparticles at the preclinical stage. The QbD approach demonstrated here can facilitate regulatory approval and industrial translation while circumventing the risk of discontinuation of clinical trials caused by inconsistencies in nanoparticle production.

## Methods

### QbD Process: Risk Assessment and Experimental Design

A risk assessment was prepared using an Ishikawa diagram to describe the QTPP of magnetic hyperthermia therapy and to define the CQAs for SPIONs. FSP was subsequently chosen to produce the SPIONs and a second Ishikawa diagram was applied to it to identify the CPPs. Failure mode and effects analysis was performed and the variables with a high-risk priority number (>15) were selected for optimization using DoE. The experimental design was constructed using MODDE (version 13, Umetrics AB, Sweden). In the first stage, a central composite orthogonal fractional factorial design was used to establish the effect of FSP process parameters on the SPION crystal size and SSA (Figure S1a). Three factors were varied at low, center, and high levels (Table S3). The iron concentration in the precursor solution was varied at 0.3, 0.5, and 0.7 mol L^–1^, the precursor flow rate was varied at 3, 6, and 9 mL min^–1^, and the dispersion gas flow rate was varied at 3, 5.5, and 8 L min^–1^. Two additional experiments were included in the design to explore the effect of higher precursor flow rates (12 and 15 L min^–1^).

The second experimental design investigated the effects of the nanoparticle size and composition of SPIONs on their magnetic and heating properties. A geometrically and mathematically balanced D-optimal design was used (Figure S1b). One qualitative factor was varied at four levels, and two quantitative factors were varied at three levels (Table S3). The composition of SPION was considered as a qualitative factor and included undoped SPIONs (γ-Fe_2_O_3_), and SPIONs doped with zinc (Zn_*x*_Fe_3–*x*_O_4_), manganese (Mn_*x*_Fe_3–*x*_O_4_), and magnesium (Mg_*x*_Fe_3–*x*_O_4_). Dopant concentration was varied at *x* = 0.25, 0.5, and 0.75, and the nanoparticle crystal size was varied at 6, 15, and 30 nm. The nanoparticle size was controlled using the model developed from the first DoE. The central composite orthogonal design used multiple linear regression for model fitting, while the D-optimal design used partial least-squares. The model fit was reviewed by examining the *R*^2^, *Q*^2^, model validity, and reproducibility. Model adequacy was further assessed by determining lack-of-fit through ANOVA analysis and inspecting the normal probability plot of residuals. The model was fine-tuned to improve predictability by removing nonsignificant model terms. A value of *p* < 0.05 was considered significant. Contour plots were constructed to visualize the effect of factors on the responses.

### Synthesis of Nanoparticles

The undoped and doped ferrites were synthesized by FSP.^100^ Liquid precursor solutions for undoped SPIONs were prepared by dissolving iron(III) nitrate nonahydrate (purity 98%; Sigma-Aldrich, Sweden) in a solvent mixture (1:1) of 2-ethylhexanoic acid (99%; Sigma-Aldrich) and ethanol (>99.7%, HPLC grade; VWR, Belgium) to obtain a total iron concentration as per the experimental design (Table 1). Doped SPIONs were synthesized by the addition of the dopant precursor, either zinc nitrate hexahydrate (purity 98%; Sigma-Aldrich), manganese(II) nitrate tetrahydrate (purity 97%; Sigma-Aldrich), or magnesium nitrate hexahydrate (purity 98%; Sigma-Aldrich). The dopant concentration was varied as per the experimental design (Table 1) to obtain a total metal concentration of 0.7 mol L^–1^. The precursor solutions were stirred for at least 1 h at room temperature. The pilot flame was ignited by a premixed supporting flame of CH_4_ and O_2_ (>99.5%, Linde AGA Gas AB) at flow rates of 1.5 and 3.2 L min^–1^, respectively. The precursor was fed to the pressure-assisted nozzle (1.6 bar) with a precision syringe pump and dispersed using O_2_ (>99.5%, Linde AGA Gas AB, Sweden) at flow rates as per the experimental design (Table 1). The doped SPIONs were prepared in three different target crystal sizes referred to as small (6 nm), mid (15 nm), and large (30 nm). It should be noted that the dopants can affect the ferrite crystal size, so the actual crystal size might deviate from the target sizes denoted here. Sheath gas at 5 L min^–1^ O_2_ was fed through the outermost sinter metal plate of the FSP burner. Gas flow rates were controlled with calibrated mass flow controllers (Bronkhorst, The Netherlands). The particles were collected on a glass fiber filter (Albert LabScience, Germany) with a Mink MM 1144 BV vacuum pump (Busch, Sweden).

The scalability of the FSP technique was tested with a pilot-scale FSP reactor at the DELVEC O.E. (Greece) on the Mn_0.25_Fe_2.75_O_4_ nanoparticles. The liquid precursor was prepared identically to the lab scale synthesis at a metal concentration of 0.7 mol L^–1^, by dissolving iron(III) nitrate nonahydrate (purity 98%; Sigma-Aldrich) and manganese(II) nitrate tetrahydrate (purity 97%; Sigma-Aldrich), in a solvent mixture (1:1) of 2-ethylhexanoic acid (99%; Sigma-Aldrich) and ethanol (>99.7%, HPLC grade; VWR, Belgium). Precursor solutions were stirred for at least 1 h at room temperature. The pilot flame was ignited by a premixed supporting flame of CH_4_ and O_2_ (>99.5%, Linde) at flow rates of 3 and 6.4 L min^–1^, respectively. The precursor was fed to the pressure-assisted nozzle (4 bar) with a precision magnetic pump (mzr-7255, HNP Mikrosysteme GmbH, Germany) and dispersed using O_2_ (>99.5%, Linde). Both the precursor solution and dispersion gas were delivered at a flow rate of 55 mL min^–1^. Sheath gas at 10 L min^–1^ O_2_ was fed through a toroidal grid of the Pilot-FSP burner. Gas flow rates were controlled with calibrated mass flow controllers (Bronkhorst, The Netherlands). The particles were collected on PTFE filters (type 9-550-3.12 WOKU Filtermedien GmbH & Co. KG, Germany) with a vacuum pump (HRD 14 T FUK-105/2,2 Elektror, GmbH, Germany).

### Citrate Coating of Nanoparticles

To perform the citrate coating, 25 mg of nanoparticle powder was first dispersed in 5 mL of Milli-Q water by sonication for 5 min at 90% amplitude using a cup horn ultrasonicator (Sonics), supplemented with a 10 s vortex mixing every 1 min. 50 mg of trisodium citrate dihydrate (Sigma-Aldrich) was added to the suspension and dissolved by magnetic stirring for 10 min. The reaction mixture was then heated to 70 °C for 30 min. Thereafter, the reaction was quenched by cooling the suspension to room temperature. The resulting product was purified from unreacted citrate by centrifugation at 17 500*g* for 20–40 min and washing with water. The washing step was performed twice, after which the citrate-coated ferrites were redispersed in Milli-Q water at a concentration of 5 mg mL^–1^.

### Characterization of Nanoparticles

Thermogravimetric analysis (TGA) was performed to determine the purity of the synthesized nanoparticle powders. Around 0.5–2 mg of powders were placed in 100 μL platinum pans and heated at 10 °C min^–1^ from room temperature to 900 °C under a dry nitrogen atmosphere. Mass losses were calculated from the TGA spectra normalized at 120 °C to exclude moisture adsorbed onto the nanoparticles. The elemental composition of the doped SPIONs was investigated by inductively coupled plasma optical emission spectroscopy (ICP-OES). To prepare the measurement sample, 2 mg of ferrite powder was dissolved in 300 μL of 37% hydrochloric acid (Sigma-Aldrich), and heated at 80 °C for 1 h. The solution was then cooled to room temperature, diluted to 50 mL with ASTM Type I water blank (SPEX), filtered using 0.45 μm filters (Cytiva), and analyzed using ICP-OES.

X-ray diffraction (XRD) patterns were measured at ambient temperature using a MiniFlex X-ray diffractometer (Rigaku Europe, Germany) with Cu Kα1 radiation (1.5406 Å) at 40 kV and 15 mA. The patterns were recorded between 10° and 80° 2θ at a step size of 0.01° and a scan speed of 2.00° min^–1^. The D/teX detector was used to suppress the iron fluorescence background. The XRD data were analyzed using PDXL2 (version 2.8, Rigaku Europe). All patterns were normalized relative to the peak intensity corresponding to the (311) crystal plane. The average crystal size (*d*_XRD_) of nanoparticles was calculated by Rietveld refinement analysis and the Scherrer equation using the PDXL2 software. The three-dimensional visualization of corresponding crystal structures was generated using the VESTA software (version 3.5),^101^ with the data from Rietveld refinement of the XRD patterns. The specific surface area (SSA) of nanoparticles was determined by nitrogen adsorption at 77 K following the Brunauer–Emmett–Teller method using a TriStar II Plus system (Micromeritics) after degassing for at least 3 h at 110 °C under a flow of nitrogen gas.

Mössbauer measurements were carried out at 295 K using a ^57^Co (Rh) source. The powder samples were mixed with boron nitride to form absorbers (Ø 8 mm) with a concentration of about 5 mg cm^–2^ of the actual substance. Calibration spectra were recorded from an iron metal foil at room temperature. The resulting spectra were analyzed using a least-squares Mössbauer fitting program. X-ray photoelectron spectroscopy (XPS) spectra were recorded on an Ulvac-Phi Quantera II XPS microprobe spectrometer by using monochromatic Al Kα radiation. The measurements were conducted under constant exposure to low-energy argon ions and electrons to prevent charge build-up. Both surveys and high-resolution core-level spectra were recorded for elements of interest and shifted using the carbon 1 s peak at 285 eV for adventitious carbon as the charge reference. The particle magnetization was recorded on a vibrating sample magnetometer (Lake Shore Cryotronics). Magnetization versus magnetic field was measured in the field range of ±1000 mT at room temperature. The saturation magnetization, coercivity, and remanence were determined from the magnetization curves.

The morphology of undoped and doped SPIONs was analyzed with a transmission electron microscope (JEOL JEM-2100F, Japan) operating at 200 kV. The samples were suspended in 99.5% ethanol and deposited as a 5 μL drop on a Formvar/Carbon 300 square mesh copper grid (Delta Microscopies, France). The particle sizes were measured by counting 100 particles using the ImageJ software, and plotted as histograms using the Sturges method.^102^ The particle size distributions of all samples were determined to be log-normally distributed using the Shapiro-Wilk test for normality and subsequently fitted to a log-normal distribution. The primary particle sizes were calculated as the geometric mean from the log-normal curve fitting. Detailed morphological analysis of Mn_0.5_Fe_2.5_O_4_ nanoparticle was carried out on a scanning transmission electron microscope (Themis Z instrument, Thermo Fisher Scientific) operated at 300 kV, to generate high-angle annular dark-field TEM images. Simultaneous energy-dispersive X-ray spectroscopy and energy-loss spectroscopy were performed using a convergence semiangle of 21.4 mrad, a collection semiangle of 23.0 mrad, and a probe current of 150 pA. The resulting spectra were combined using hypermodal data fusion.^71,72^ 4D scanning diffraction was performed in nanobeam diffraction mode with a convergence angle of 0.5 mrad, a probe current of 10 pA, and a scan rate of approximately 300 frames/s. Bragg spot indexing, crystallographic orientation maps, and the data for the Bragg vector map were extracted using the py4DSTEM software package.^73^ The citrate coating of the nanoparticle was investigated using Fourier transform infrared spectroscopy (FTIR). The FTIR spectra were obtained in the range of 400–4000 cm^–1^ with an α II spectrometer (Bruker, Germany) equipped with a platinum ATR accessory. The intensity-weighted hydrodynamic diameter and ζ-potential of citrate-coated SPIONs were measured using dynamic light scattering (Litesizer 500, Anton Paar GmbH, Austria) in backscattering geometry at 20 °C. Prior to measurement, the suspensions were diluted to a concentration of 0.1 mg mL^–1^.

### Heat Dissipation Measurement

Hyperthermia measurements were performed on a 5 mg mL^–1^ citrate-coated nanoparticle suspension. The thermal dissipation of nanoparticle suspensions was measured using an oscillating magnetic field apparatus (MagneTherm; Nanotherics Ltd., U.K.). One mL of the ferrite suspension was transferred to a 2 mL glass vial and placed inside a 9-turn coil. The nominal oscillation frequency was set to 592.2 kHz and the magnetic field strength to 14 mT. Suspension temperature was measured with a fiber optic probe every second for 5 min. The heat dissipation was evaluated by calculating the intrinsic loss power using eqs 1 and 2.
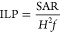
1
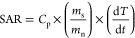
2where, *H* and *f* are the amplitude and frequency of AMF, respectively, SAR is the specific absorption rate, d*T*/d*t* is the initial slope of the heating curve, *m*_s_ is the mass of the sample, *m*_n_ is the mass of the nanoparticle, and *C*_p_ is the specific heat capacity of the sample.^78^ The hyperthermia performance of uncoated nanoparticles was also measured in dimethyl sulfoxide (DMSO; Sigma-Aldrich) at a concentration of 3 mg mL^–1^.

### Cell Culture

Cell culture media and reagents were purchased from Thermo Fisher Scientific or Sigma-Aldrich. All cells were originally obtained either from the American Type Culture Collection (Caco-2, MDCK, SW-480, and HT-29) or Thermo Fisher Scientific (HEK-293; Waltham, MA). Caco-2, SW-480, and HT-29 cells, at passages 95–105, 109, and 50, respectively, were maintained in Dulbecco’s modified Eagle’s medium containing 10% (v/v) fetal bovine serum, 1% (v/v) penicillin/streptomycin solution (10 mg mL^–1^), and 1% (v/v) nonessential amino acids. Caco-2 cells were additionally cultured without a penicillin/streptomycin solution for an *in vitro* hyperthermia study. HEK-293 cells (Flp-In-293 control cells),^103^ passage 16, were maintained in high glucose Dulbecco’s modified Eagle’s medium containing 10% (v/v) fetal bovine serum, 1% (v/v) penicillin/streptomycin solution (10 mg mL^–1^), and 1% (v/v) l-glutamine. All cells were cultured at 37 °C in a humidified incubator in 75 cm^2^ tissue culture flasks. The Caco-2, SW-480, and HT-29 cells were cultured at 10% CO_2_, while the HEK-293 cells were cultured at 5% CO_2_.

### Cytotoxicity of Nanoparticles

Cell viability was measured to assess the cytotoxicity of the citrate-coated midsized γ-Fe_2_O_3_, Zn_0.5_Fe_2.5_O_4_, Mn_0.5_Fe_2.5_O_4_, Mg_0.5_Fe_2.5_O_4_, and Mn_0.25_Fe_2.75_O_4_ nanoparticles. The stock suspension of 6 mg mL^–1^ citrate-coated ferrites was prepared as described above. The stock suspensions were then diluted with the corresponding cell culture medium to achieve nanoparticle concentrations of 600, 400, 200, and 100 μg mL^–1^. The cells were plated into black and opaque 96-well plates, at a density of 5 × 10^4^ cells per well in 250 μL of culture medium. The cells were allowed to attach to the plate for 24 h before being incubated with the citrate-coated nanoparticles. Thereafter, the cell culture medium was replaced by 100 μL of nanoparticle suspensions in four replicates and incubated for 24 h. Cell culture medium (without nanoparticles) was used as a negative control, while positive controls were prepared by incubating the cells in the culture medium containing 0.2% (v/v) Triton X-100 (Sigma-Aldrich). Cell viability was measured by the CellTiter-Glo Luminescent assay (Promega), according to the manufacturer’s instructions. The luminescence signal of each well was determined with a plate reader (Tecan, Switzerland).

### *In Vitro* Magnetic Hyperthermia and Cellular Uptake

*In vitro* magnetic hyperthermia studies were performed on Caco-2 cells using ferrite nanoparticles optimized with QbD. The cells were seeded onto 35 mm Petri dishes (Sarstedt AG, Germany) at a density of 3 × 10^5^ cells per dish in 2 mL of culture medium. The cells were allowed to attach and grow for 2–3 days until 70–80% confluency. The medium was changed every second day. The cellular hyperthermia treatment was performed using aqueous suspensions of citrate-coated nanoparticles (γ-Fe_2_O_3_ and Mn_0.25_Fe_2.75_O_4_). The stock suspensions (5 mg mL^–1^) were diluted with cell culture medium without fetal bovine serum and phenol red to achieve a final concentration of 400 μg mL^–1^. The cells were treated in triplicate with the SPION suspension (2 mL) and incubated for 2 h at 37 °C and 10% CO_2_. After incubation, the cells were exposed to AMF (14 mT, 592.2 kHz) for 30 min. Positive controls were prepared by adding 10% (v/v) Triton X-100 to the cells to achieve a final concentration of 0.2% (v/v) in the cell culture medium. The cell culture medium without SPION suspension was used as a negative control. Cell culture medium (50 μL) was collected from cells treated with SPIONs before and after the AMF exposure, and from negative and positive controls. Cell death caused by magnetic hyperthermia was measured in the collected aliquots by using the LDH-Glo cytotoxicity assay (Promega). The luminescence signal from the aliquots was determined using a plate reader (Tecan, Switzerland).

The uptake of nanoparticles in the Caco-2 cells was investigated using ICP-OES. Stock suspensions (6 mg mL^–1^) of citrate-coated midsized γ-Fe_2_O_3_ and Mn_0.25_Fe_2.75_O_4_ nanoparticles were prepared, and diluted with culture medium to 400 μg mL^–1^ (final concentration). Caco-2 cells were cultured as described above and plated in 24-well transparent plates at a density of 2 × 10^5^ cells per well in 800 μL of culture medium. The cells were allowed to attach for 24 h (at 37 °C and 10% CO_2_) before treatment with the nanoparticles. Subsequently, the cell culture medium was replaced with 800 μL of the nanoparticle suspensions (400 μg mL^–1^), and the cells were incubated for 2 h (at 37 °C, 10% CO_2_) in six replicates. Cell culture medium (without nanoparticles) was used as a negative control. After the incubation period, the culture medium containing the nanoparticles was removed, and the cells were washed twice with 1 mL of preheated phosphate-buffered saline (PBS) to remove excess nanoparticles. To prepare the samples for ICP-OES, the cells were trypsinized, transferred to centrifuge tubes, and centrifuged at a speed of 400*g* for 5 min. Then, the supernatant was removed and 0.5 mL of 12 M HCl (Sigma-Aldrich) was added to the pelleted cells, and the mixture was heated to 80 °C for 1 h to digest the cells and dissolve the nanoparticles. The resulting HCl solutions were then analyzed by using ICP-OES.

### Statistical Analysis

A two-way analysis of variance (ANOVA) using Tukey’s multiple comparison test was used to compare the groups in cell viability assay and cellular hyperthermia experiments. Data analysis was performed using GraphPad Prism 9.0 software (La Jolla, CA). *p* values were calculated as >0.05 (ns), ≤0.05 (*), ≤0.01 (**), ≤0.001 (***), and <0.0001 (****).

## Supporting Information Available

Illustration of DoE design of central composite orthogonal and D-optimal design (Figure S1); TEM images and particle size distribution of undoped SPIONs (Figure S2); plots of observed vs predicted crystal size and SSA (Figure S3); XRD patterns and detailed XPS spectra of nanoparticles (Figures S4, S5, and S6); energy-dispersive X-ray spectra, Bragg vector map, and crystal orientation map obtained from scanning TEM analysis (Figure S7); magnetization curves of doped SPIONs (Figure S8); FTIR spectra of citrate-coated SPIONs (Figure S9); viability of SW-480 and HT-29 cells after exposure to undoped and doped ferrite nanoparticles (Figure S10); comparison of different SPION synthesis methods (Table S1); failure mode and effects analysis (Table S2); DoE design space (Table S3); summary of synthesis conditions and physicochemical properties of γ-Fe_2_O_3_ nanoparticles (Table S4); Mössbauer parameters of nanoparticles (Table S5); adjusted model for crystal size and SSA (Table S6); summary of synthesis conditions, physicochemical, magnetic, and heating characteristics of undoped and doped nanoparticles (Tables S7 and S8); adjusted model for ILP of SPIONs measured in water and DMSO (Table S9) (PDF)

## Abbreviations

AMF: alternating magnetic field
CPP: critical process parameters
CQA: critical quality attributes
DoE: design of experiments
FSP: flame spray pyrolysis
ILP: intrinsic loss power
QbD: quality by design
QTPP: quality target product profile
SPION: superparamagnetic iron oxide nanoparticle
SSA: specific surface area
TEM: transmission electron microscopy
XPS: X-ray photoelectron spectroscopy
XRD: X-ray diffraction
