# Supplementary material for: Pharmaceutical Quality by Design Approach to Develop High-Performance Nanoparticles for Magnetic Hyperthermia
Source: ACS Nano. 2024 May 30;18(23):15284–302. doi: 10.1021/acsnano.4c04685 (PMC11171760; doi:10.1021/acsnano.4c04685)
Supplement: Supplementary file 1 — nn4c04685_si_001.pdf [file nn4c04685_si_001.pdf]

Supporting Information

Pharmaceutical quality by design approach to develop high-performance nanoparticles for magnetic hyperthermia

*Shaquib Rahman Ansari[1], Yael del Carmen Suárez-López[1], Thomas Thersleff[2], Lennart Häggström[3], Tore Ericsson[3], Ioannis Katsaros[4], Michelle Åhlén[4], Maria Karlgren[5], Peter Svedlindh[4], Carlos M. Rinaldi-Ramos[6], and Alexandra Teleki[1]**

[1]Department of Pharmacy, Science for Life Laboratory, Uppsala University, 75123 Uppsala, Sweden

[2]Department of Materials and Environmental Chemistry, Stockholm University, 10691 Stockholm, Sweden

[3]Department of Physics and Astronomy, Uppsala University, 75121 Uppsala, Sweden

[4]Department of Materials Science and Engineering, Uppsala University, 75103 Uppsala, Sweden

[5]Department of Pharmacy, Uppsala University, 75123 Uppsala, Sweden

[6]Department of Chemical Engineering and J. Crayton Pruitt Family Department of Biomedical Engineering, University of Florida, Gainesville, Florida 32611-6005, USA

*Email: alexandra.teleki@scilifelab.uu.se

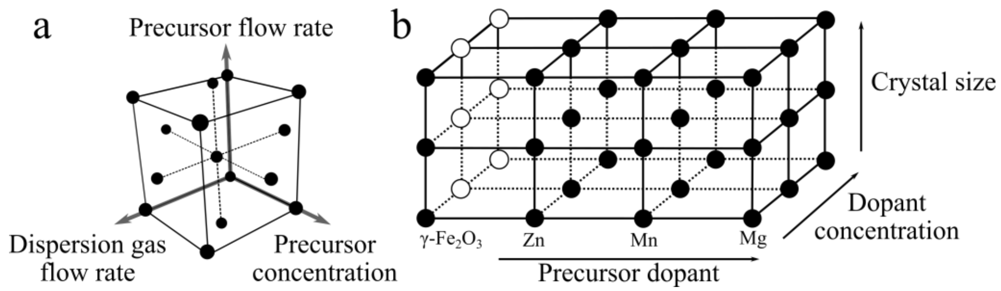

Figure S1. Graphical representation of the design space explored through design of experiments (DoE). (a) Central composite orthogonal design used for optimization of SPION size, and (b) D-optimal design used for optimization of magnetic hyperthermia.

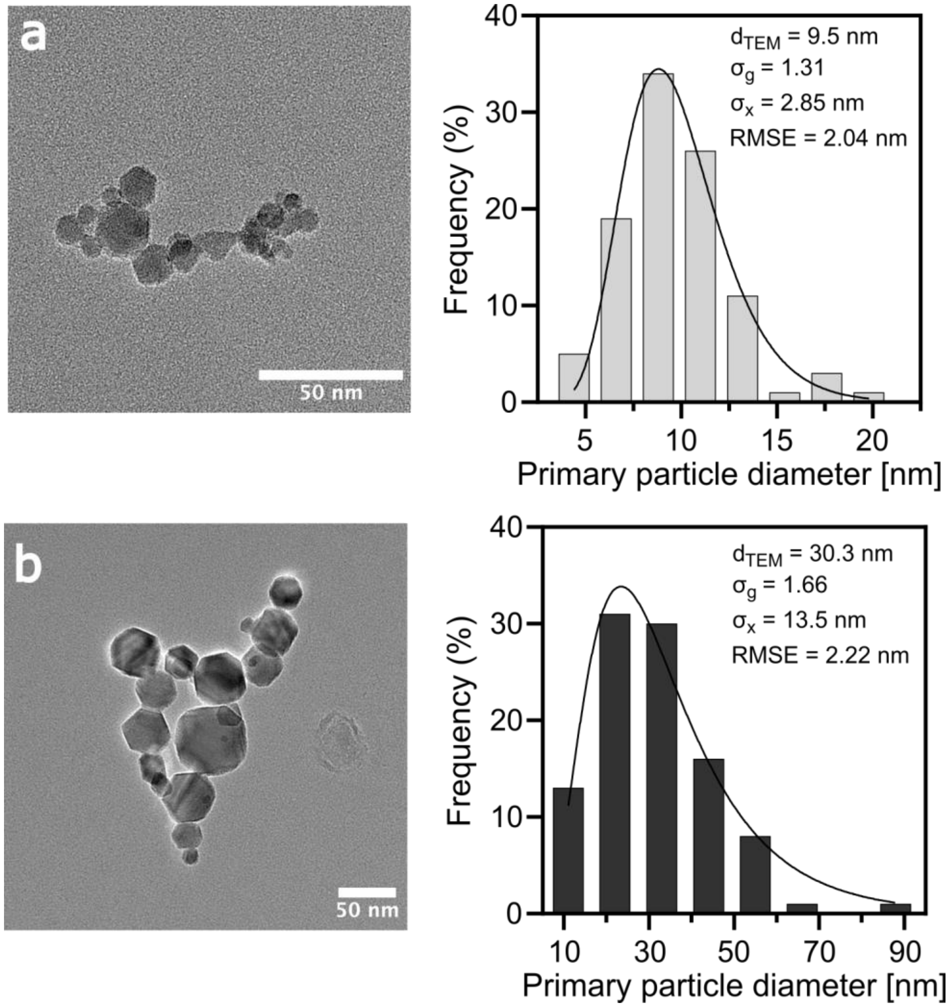

Figure S2. Representative TEM images and corresponding particle size distribution of γ-Fe_2O_3 nanoparticles with crystal sizes (d_{XRD}) of (a) 6 nm and (b) 29.6 nm. (σ_x, arithmetic standard deviation; RMSE, root mean squared error).

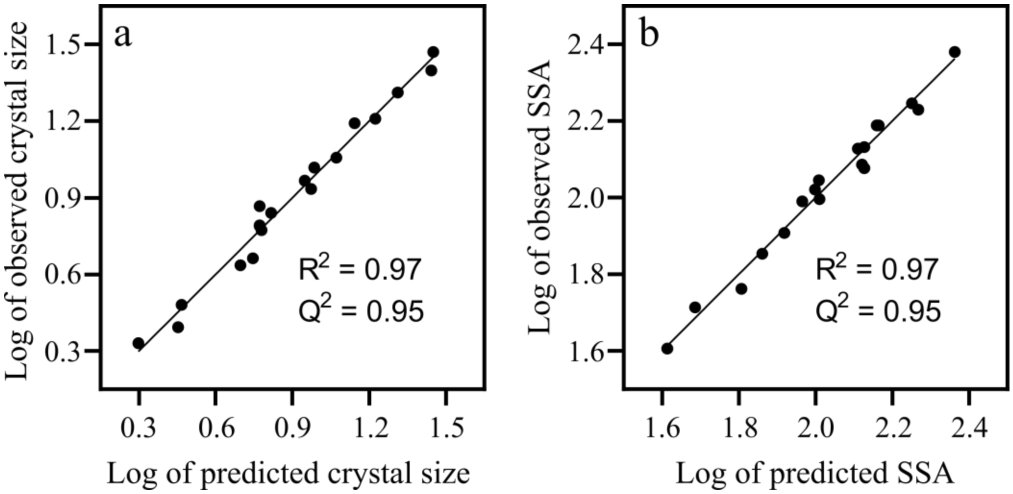

Figure S3. Correlation between the measured and the predicted values of a) crystal size and b) specific surface area (SSA) of undoped SPIONs after modeling.

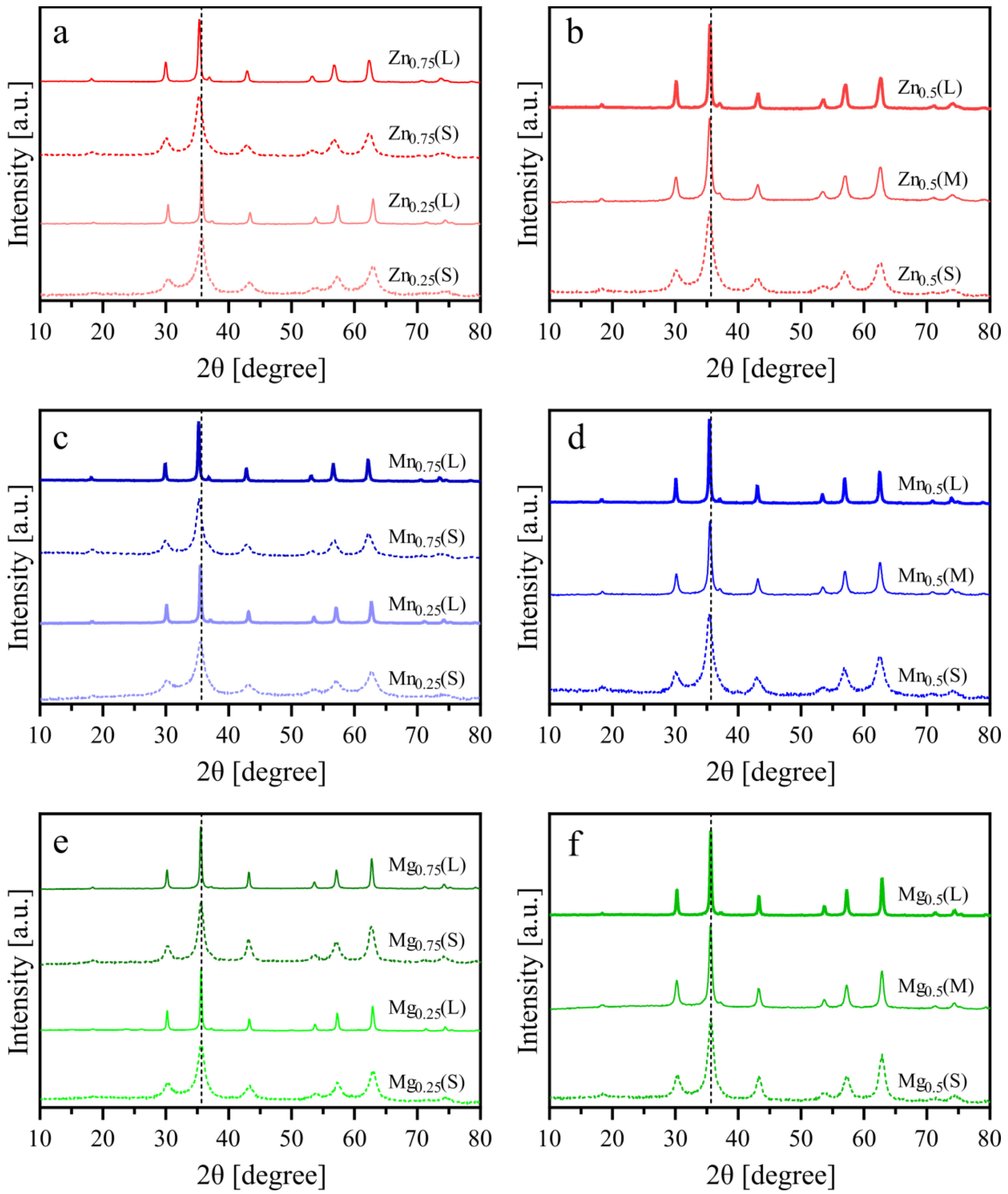

Figure S4. XRD patterns of (a,b) Zn-doped SPIONs, (c,d) Mn-doped SPIONs, and (e,f) Mg-doped SPIONs. Target crystal sizes of SPIONs are indicated by letters S (small), M (mid), and L (large).

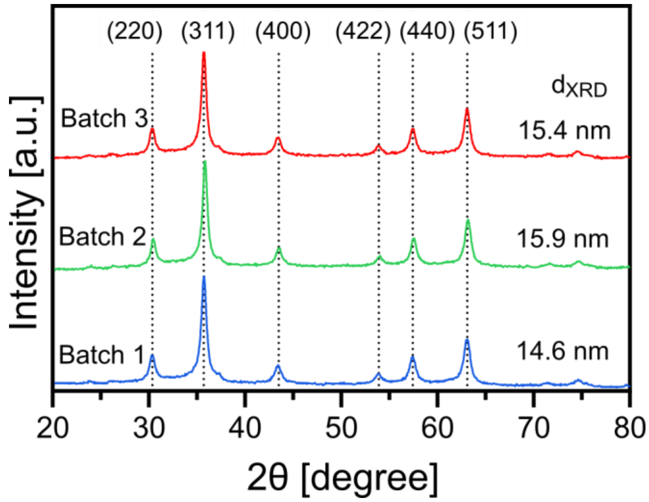

Figure S5. X-ray diffraction patterns of three batches of γ-Fe_2O_3 nanoparticles. The batches were made using identical FSP conditions of 0.7 M precursor concentration, 6 mL min^{-1} precursor flow rate, and 3 L min^{-1} dispersion gas flow rate.

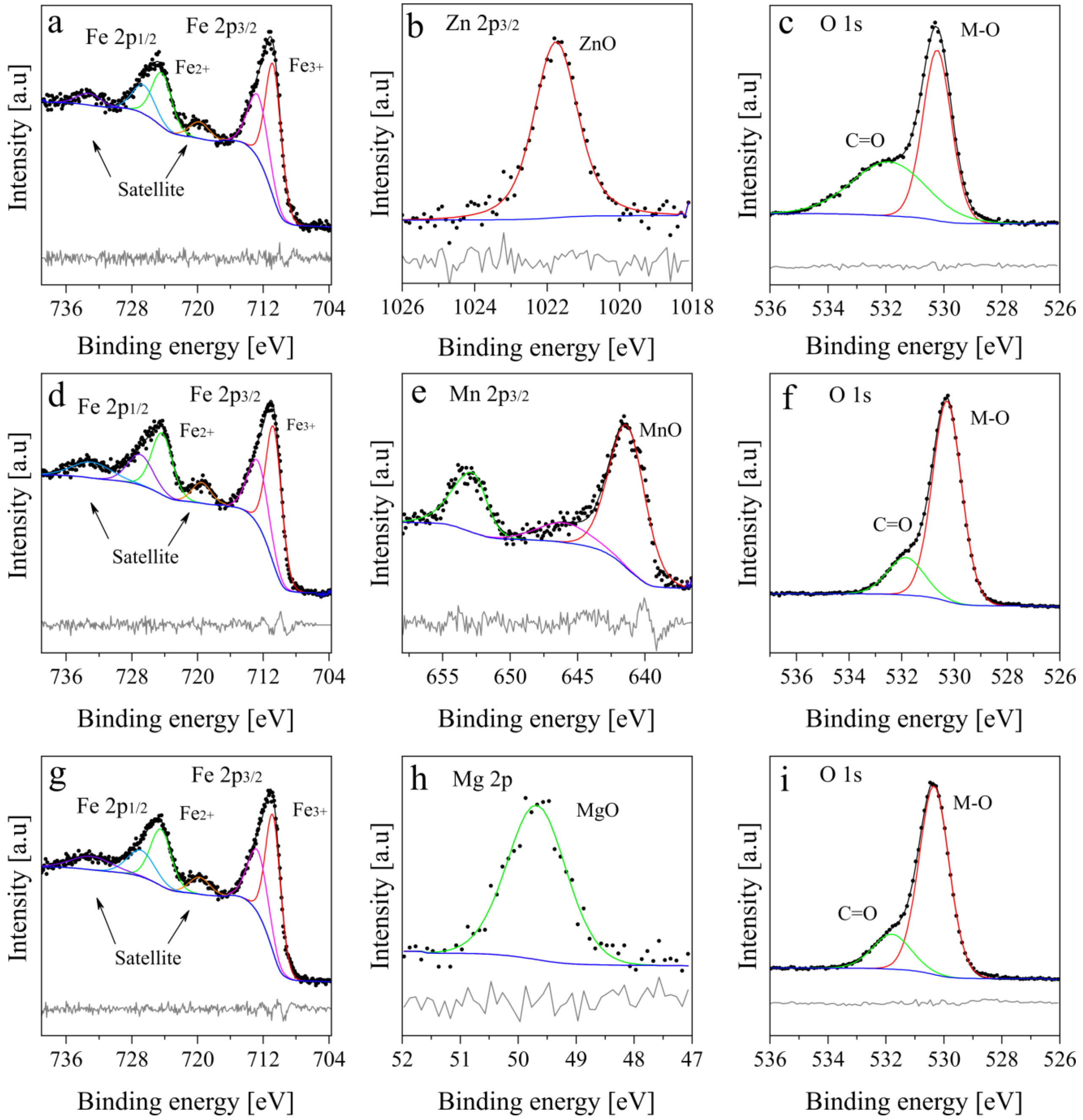

Figure S6. XPS patterns of midsized (a-c) $Zn_{0.5}Fe_{2.5}O_4$, (d-f) $Mn_{0.5}Fe_{2.5}O_4$, and (g-i) $Mg_{0.5}Fe_{2.5}O_4$ nanoparticles.

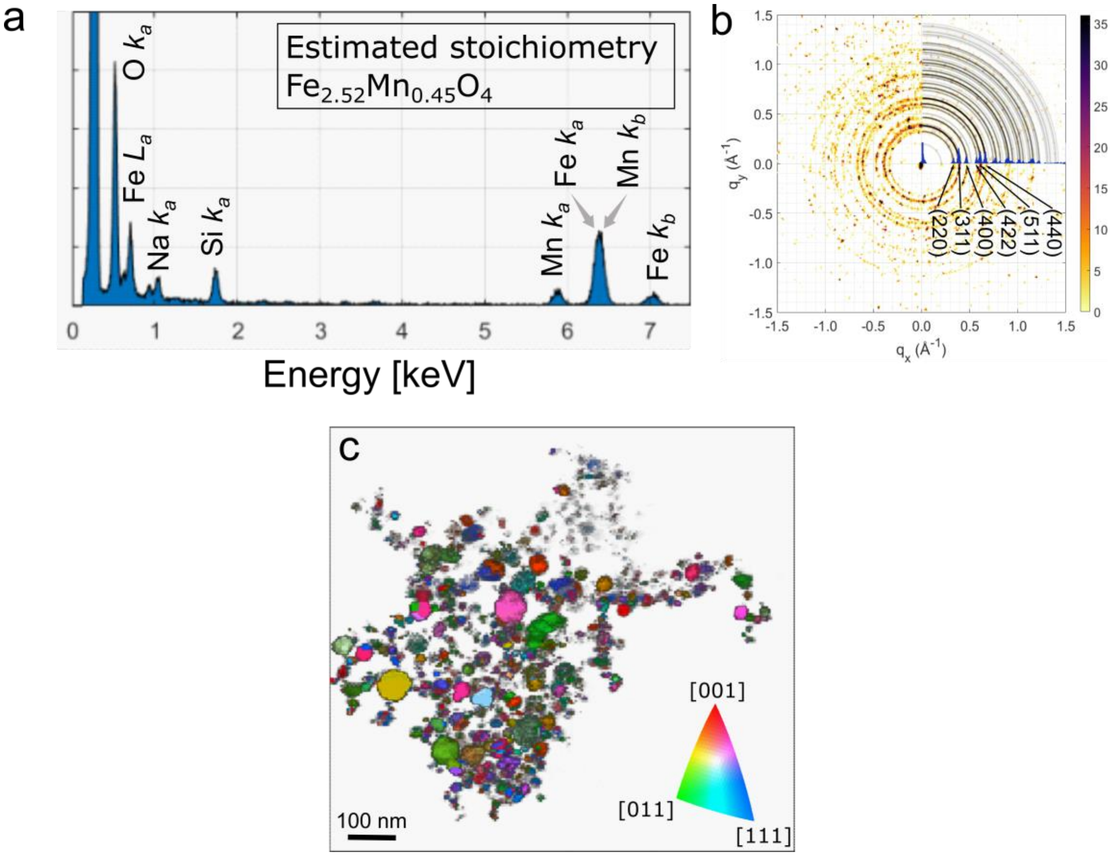

Figure S7. Chemical composition and crystal structure of $Mn_{0.5}Fe_{2.5}O_4$ nanoparticles. (a) Energy dispersive X-ray spectra. (b) Bragg vector map from a collection of particles overlaid with a simulated ring diffraction pattern of $Mn_{0.5}Fe_{2.5}O_4$ nanoparticles. (c) Out-of-plane crystal orientation map obtained from STEM analysis.

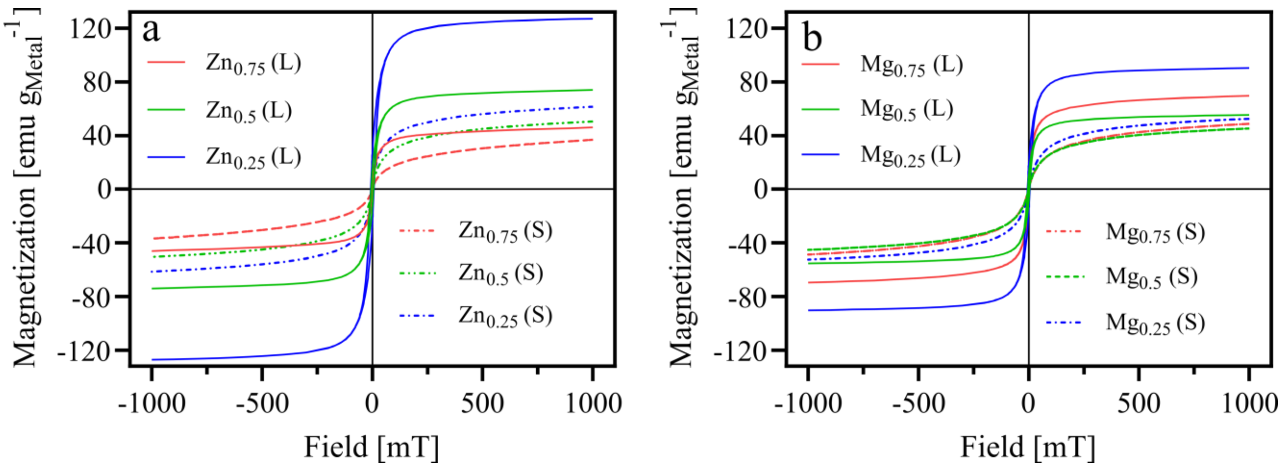

Figure S8. Magnetization curves at 300 K of small (solid line) and large-sized (dotted line) nanoparticles of different compositions. (a) $Zn_{0.25}Fe_{2.75}O_4$ (blue), $Zn_{0.5}Fe_{2.5}O_4$ (green), and $Zn_{0.75}Fe_{2.25}O_4$ (red); and (b) $Mg_{0.25}Fe_{2.75}O_4$ (blue), $Mg_{0.5}Fe_{2.5}O_4$ (green), and $Mg_{0.75}Fe_{2.25}O_4$ (red).

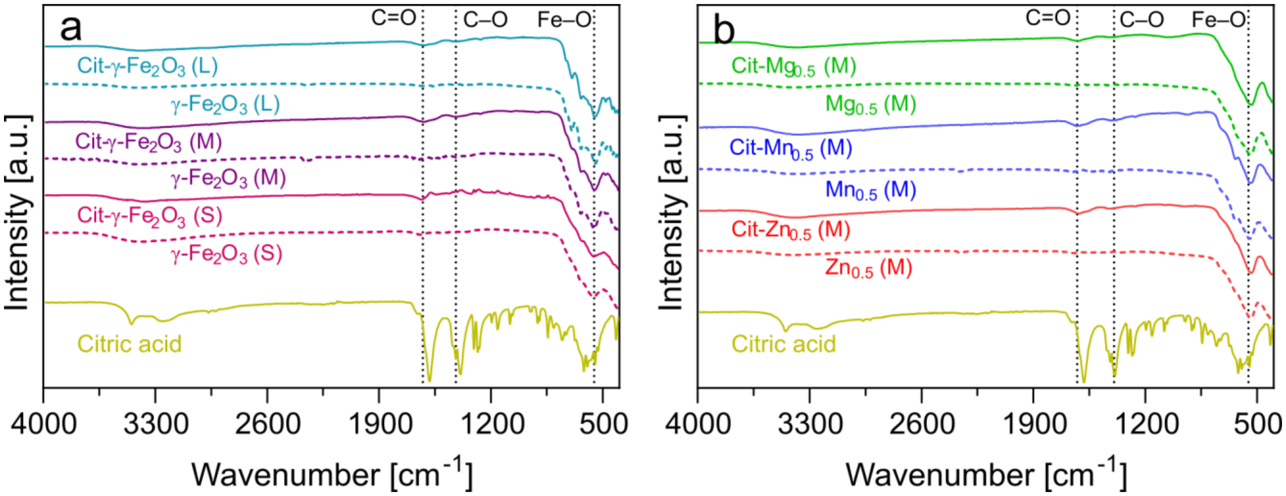

Figure S9. FTIR spectra of uncoated (dashed lines) and citrate coated (solid lines) (a) undoped, and (b) doped SPIONs.

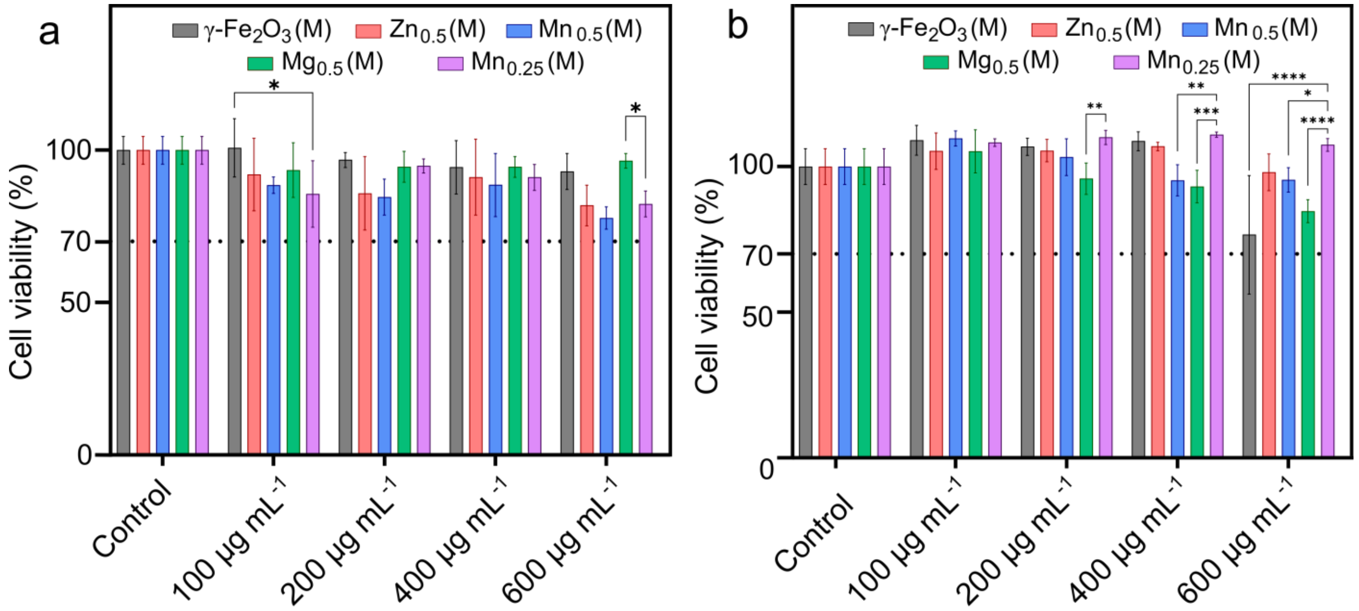

Figure S10. Cell viability of nondifferentiated (a) SW-480 and (b) HT-29 cell lines after exposure to mid-sized γ-Fe_2O_3, $Zn_{0.5}Fe_{2.5}O_4$, $Mn_{0.5}Fe_{2.5}O_4$, $Mg_{0.5}Fe_{2.5}O_4$, and $Mn_{0.25}Fe_{2.75}O_4$ nanoparticles at different concentrations (100, 200, 400, and 600 µgmL^{-1}). Cell viability was determined using the CellTiter-Glo luminescent cell viability assay and calculated as a percentage of the control. Data shows the average of at least four experiments ± SD. $p < 0.1234$ (ns), 0.0332 (*), 0.0021 (**), 0.0002 (***), and 0.0001 (****).

Table S1. Comparison of different methods to synthesize SPIONs.[1,2]

Method	Complexity	Process time	Size control	Size distribution	Morphology	Crystallinity	Yield	Scalability
Thermal decomposition	Very complicated	Hours-days	Very good	Very narrow	Cube-sphere	High	Medium	Medium
Co-precipitation	Very Simple	Minutes-hours	Bad	Very broad	Irregular sphere	Low	High	High
Flame spray pyrolysis	Very simple	Minutes	Good	Moderately narrow	Hexagonal-sphere	High	Very high	Very high

Table S2. Assignment of Risk Priority Number (RPN) to the factors affecting flame synthesis of SPIONs using the failure mode and effects analysis.

Factor	Severity[a] (S)	Occurrence[b] (O)	Detectability[c] (D)	RPN (S x O x D)
Precursor metal concentration	3	2	3	**18**
Precursor solution composition	4	1	3	12
Metal precursor salt	2	1	3	6
Solvent	2	1	3	6
Precursor solution feed flow rate	4	2	3	**24**
Ignition gas flow rate	4	1	2	8
Ignition gas composition	4	1	2	8
Dispersion gas flow rate	4	1	4	**16**
Dispersion gas composition	3	1	1	3
Sheath gas flow rate	1	1	1	1
Sheath gas composition	2	1	1	2
Nozzle tip pressure	4	1	3	12
Environment temperature	1	1	4	4
Environment air quality	1	1	4	4
Humidity	1	1	4	4
Reducing environment	3	1	4	12
Nozzle design	2	1	2	4
Open/closed flame	3	1	4	12
Capillary angle	2	2	3	12
Capillary dimensions	3	1	4	12
Nanoparticle collection	2	1	4	8

[a]Severity is a measure of the effect of change in a parameter on the product quality. [b]Occurrence is the likelihood of a parameter to get affected. [c]Detectability indicates the ease of detecting the changes to a parameter. The factors were ranked on severity, occurrence, and detectability as 4 (high), 3 (moderate), 2 (low), and 1 (none). Factors with RPN > 15 are indicated in **bold**.

Table S3. DoE design space showing the factors investigated in size optimization, and magnetic hyperthermia optimization of SPIONs.

SPION crystal size optimization design			
Factor	Low level	Center level	High level
Iron concentration [mol L^{-1}]	0.3	0.5	0.7
Precursor flow rate [mL min^{-1}]	3	6	9
Dispersion gas flow rate [L min^{-1}]	3	5.5	8
**Magnetic hyperthermia optimization design**			
Dopant type	-	-	-
γ-Fe_2O_3			
$Zn_xFe_{3-x}O_4$			
$Mn_xFe_{3-x}O_4$			
$Mg_xFe_{3-x}O_4$			
Dopant concentration (x)	0.25	0.5	0.75
Crystal size [nm]	6	15	30

Table S4. Summary of synthesis conditions, physicochemical properties, and thermal stability of undoped SPIONs prepared according to the central composite orthogonal design.

Exp No	Precursor flow rate [mL min^{-1}]	Precursor concentration [mol L^{-1}]	Dispersion gas flow rate [L min^{-1}]	d_{XRD} [nm]	Weight loss (wt%)	Lattice constant (a) [Å]	SSA [m^2 g^{-1}]
1	3	0.7	3	9.3		8.341	105.0
2	3	0.7	8	3.0		8.392	176.2
3	8	0.7	3	20.5		8.342	57.8
4	8	0.7	8	10.4		8.336	99.2
5	5	0.5	5.5	7.4		8.350	119.5
6	8	0.3	8	6.9		8.332	121.9
7	8	0.3	3	15.5		8.341	80.9
8	3	0.3	8	2.1		8.453	240.2
9	3	0.3	3	5.9	4.73	8.343	134.2
10	2	0.5	5.5	2.5		8.437	169.5
11	9	0.5	5.5	11.4		8.344	97.7
12	6	0.5	2.1	16.2	3.57	8.346	71.5
13	6	0.5	8.9	4.3		8.364	154.4
14	6	0.23	5.5	4.6		8.373	154.4
15	6	0.77	5.5	8.6		8.344	111.0
16	12	0.7	3	25.0		8.342	51.7
17	15	0.7	3	29.6	2.24	8.348	40.4
18	5	0.5	5.5	6.2		8.348	135.6
19	5	0.5	5.5	6.2		8.360	136.6

Table S5. Mössbauer parameters of the γ-Fe_2O_3 and $Mn_{0.5}Fe_{2.5}O_4$ nanoparticles at 295K showing the values of the hyperfine field (B), the isomer shift (δ), quadrupole splitting (Δ), electric quadrupole shift (ε), individual Lorentzian line width (Γ) at full width at half maximum (FWHM), and the spectral intensity (I) of the components. All δ-values are given with respect to metallic α-iron at room temperature. The hyperfine results for the magnetic part (90%) in the sample 15 nm are averaged values from three different six-line patterns. Uncertainties are given in parentheses.

Nanoparticle		B [T]	δ [mm s^{-1}]	Δ [mm s^{-1}]	ε [mm s^{-1}]	Γ [mm s^{-1}]	I (%)
Undoped (6 nm)		-	0.33(2)	0.70(4)	-	0.78(6)	100
Undoped (15.9 nm)		45(1)	0.34(1)	-	-0.01(2)	0.75(3)	90(2)
			0.33(2)	0.70(2)	-	0.70(3)	10(2)
Undoped (29.6 nm)		49(1)	0.32(1)	-	0.00(1)	0.71(3)	100
$Mn_{0.5}Fe_{2.5}O_4$ (mid-sized)	A-site	47(1)	0.33(2)	-	0.00(2)	0.47(7)	38(4)
	B-site	43(1)	0.55(5)	-	0.00(2)	1.1(1)	54(5)
	Doublet	-	0.28(7)	0.4(2)	-	0.6(2)	8(2)

Table S6. Adjusted models showing the estimated coefficients and confidence intervals (95%) for crystal size and specific surface area. The models were derived using multiple linear regression.

	Crystal size		Specific surface area	
Model terms	Coefficient	CI (±)	Coefficient	CI (±)
Precursor flow rate (Pre-flow)	**0.345**[b]	0.096	**-0.222**[b]	0.060
Precursor concentration	0.084	0.034	-0.056	0.021
Dispersion gas flow rate (Dis-flow)	-0.147	0.060	0.097	0.037
Pre-flow * Pre-flow	-0.159	0.091	0.032	0.056
Dis-flow * Dis-flow	≤0.05	0.045	-0.039	0.028
Pre-flow * Dis-flow	0.094	0.092	-0.030	0.057
R^2	0.97		0.972	
Q^2	0.95		0.955	
Validity	0.80		0.87	
Reproducibility	0.98		0.97	

All responses were log transformed. [b]**Bold**: factors with the most influence on the given response. CI: confidence intervals

Table S7. Summary of synthesis conditions and physicochemical properties of undoped and doped SPIONs prepared according to the D-optimal design.

Exp No	Precursor composition	Precursor flow rate [mL min-1]/ dispersion gas flow rate [L min-1]	Fe/Dopant atomic ratio		Weight loss (%)	Crystal size [nm]	Lattice constant [Å]	SSA [m^2 g^{-1}]
			Calculated	Observed				
1	γ-Fe_2O_3	4/5.6	-	-		6	8.344	141.4
2	γ-Fe_2O_3	6/3	-	-		14.6	8.342	81.2
3	γ-Fe_2O_3	6/3	-	-		15.9	8.337	77.7
4	γ-Fe_2O_3	6/3	-	-		15.4	8.344	76.0
5	γ-Fe_2O_3	15/3	-	-		29.6	8.347	40.4
6	$Zn_{0.25}Fe_{2.75}O_4$	4/5.6	0.091	0.087		6.4	8.371	140.6
7	$Zn_{0.25}Fe_{2.75}O_4$	15/3	0.091	0.089		26.1	8.364	48.9
8	$Zn_{0.5}Fe_{2.5}O_4$	4/5.6	0.200	0.204		6.2	8.410	124.7
9	$Zn_{0.5}Fe_{2.5}O_4$	6/3	0.200	0.201	2.85	13.4	8.404	74.4
10	$Zn_{0.5}Fe_{2.5}O_4$	15/3	0.200	0.193		21.1	8.395	44.1
11	$Zn_{0.75}Fe_{2.25}O_4$	4/5.6	0.333	0.325		6.8	8.434	146.6
12	$Zn_{0.75}Fe_{2.25}O_4$	15/3	0.333	0.321		20.9	8.431	44.4
13	$Mn_{0.25}Fe_{2.75}O_4$	4/5.6	0.091	0.087		5.8	8.348	120.3
14	$Mn_{0.25}Fe_{2.75}O_4$	15/3	0.091	0.090		32.9	8.354	33.1
15	$Mn_{0.5}Fe_{2.5}O_4$	4/5.6	0.200	0.200		7	8.382	148.9
16	$Mn_{0.5}Fe_{2.5}O_4$	6/3	0.200	0.185	2.57	17	8.376	72.8
17	$Mn_{0.5}Fe_{2.5}O_4$	6/3	-	-		17.9	-	-
18	$Mn_{0.5}Fe_{2.5}O_4$	15/3	0.200	0.201		32.7	8.366	42.0
19	$Mn_{0.75}Fe_{2.25}O_4$	4/5.6	0.333	0.343		7.6	8.387	148.0
20	$Mn_{0.75}Fe_{2.25}O_4$	15/3	0.333	0.329		32.5	8.383	42.0
21	$Mg_{0.25}Fe_{2.75}O_4$	4/5.6	0.091	0.087		6.9	8.381	152.1
22	$Mg_{0.25}Fe_{2.75}O_4$	15/3	0.091	0.088		34.3	8.378	40.2
23	$Mg_{0.5}Fe_{2.5}O_4$	4/5.6	0.200	0.183		8	8.427	149.9
24	$Mg_{0.5}Fe_{2.5}O_4$	6/3	0.200	0.188	4.05	16.8	8.416	76.5
25	$Mg_{0.5}Fe_{2.5}O_4$	15/3	0.200	0.185		32.5	8.412	38.5
26	$Mg_{0.75}Fe_{2.25}O_4$	4/5.6	0.333	0.331		8.4	8.455	153.1
27	$Mg_{0.75}Fe_{2.25}O_4$	15/3	0.333	0.331		30.2	8.443	40.5

Table S8. Summary of colloidal, magnetic and heating properties of undoped and doped SPIONs prepared according to the D-optimal design.

Exp No	Precursor composition	Saturation magnetization (M_s) [emu g_{Metal}^{-1}]	Remanence (B_r) [emu g_{Metal}^{-1}]	Coercivity (H_c) [mT]	Hydrodynamic diameter (after coating) [nm]	PDI (after coating) (%)	ζ-potential [mV]		Intrinsic loss power (ILP) [nH m^2 kg^{-1}]
							Before coating	After coating	
1	γ-Fe_2O_3	49.50	0.31	0.24	357.2	25.7	33.6	-24.8	0.152
2	γ-Fe_2O_3	78.41	6.01	2.49	203	26.4	38.1	-32.6	0.407
3	γ-Fe_2O_3	72.13	6.93	3.33	146.5	14.2			0.413
4	γ-Fe_2O_3	69.20	5.68	2.68	153	21.7			0.461
5	γ-Fe_2O_3	111.90	29.38	14.16	724.7	32.4	54	-14.2	0.163
6	$Zn_{0.25}Fe_{2.75}O_4$	61.39	0.20	0.11	121.4	25.5			0.225
7	$Zn_{0.25}Fe_{2.75}O_4$	127.15	25.15	7.06	3607	11.2			0.664
8	$Zn_{0.5}Fe_{2.5}O_4$	50.52	0.15	0.13	134.2	24.3			0.068
9	$Zn_{0.5}Fe_{2.5}O_4$	64.24	3.52	1.16	131.6	23.1	28.5	-28.5	1.422
10	$Zn_{0.5}Fe_{2.5}O_4$	73.98	12.78	5.34	640.8	26.7			1.047
11	$Zn_{0.75}Fe_{2.25}O_4$	36.89	0.09	0.15	212.5	27.2			0.062
12	$Zn_{0.75}Fe_{2.25}O_4$	46.02	5.69	2.98	1659.2	32.1			0.633
13	$Mn_{0.25}Fe_{2.75}O_4$	45.99	0.17	0.13	110.4	25.9			0.179
14	$Mn_{0.25}Fe_{2.75}O_4$	114.12	24.99	7.35	5227	326			0.446
15	$Mn_{0.5}Fe_{2.5}O_4$	54.97	0.19	0.16	86.9	24.7			0.278
16	$Mn_{0.5}Fe_{2.5}O_4$	88.20	5.84	1.65	114.9	22	37.4	-22.7	1.896
17	$Mn_{0.5}Fe_{2.5}O_4$	91.15	5.41	1.79					1.922
18	$Mn_{0.5}Fe_{2.5}O_4$	97.71	20.76	6.83	2289	26.5			0.404
19	$Mn_{0.75}Fe_{2.25}O_4$	51.86	0.19	0.13	95.8	24.7			0.179
20	$Mn_{0.75}Fe_{2.25}O_4$	97.27	18.78	6.03	3990	24.3			0.583
21	$Mg_{0.25}Fe_{2.75}O_4$	52.49	0.17	0.15	96.3	24.2			0.090
22	$Mg_{0.25}Fe_{2.75}O_4$	90.23	17.15	5.51	4412	158			0.840
23	$Mg_{0.5}Fe_{2.5}O_4$	45.29	0.12	0.15	135	26.4			0.054
24	$Mg_{0.5}Fe_{2.5}O_4$	62.13	1.16	0.36	3968	28.7	0.5	-22.5	0.821
25	$Mg_{0.5}Fe_{2.5}O_4$	55.24	8.84	3.91	4531	25.6			1.112
26	$Mg_{0.75}Fe_{2.25}O_4$	48.74	0.11	0.16	184	23.3			0.060
27	$Mg_{0.75}Fe_{2.25}O_4$	69.66	8.45	2.76	688.3	23			0.730

Table S9. Adjusted model showing the estimated coefficients and confidence intervals (95%) for intrinsic loss power (ILP) of SPIONs measured in water and DMSO. The models were derived using partial least squares regression.

	ILP measured in water		ILP measured in DMSO	
Model terms	Coefficient	CI (±)	Coefficient	CI (±)
$Zn_xFe_{3-x}O_4$	0.014	0.155	-0.035	0.113
$Mn_xFe_{3-x}O_4$	0.097	0.132	0.018	0.107
$Mg_xFe_{3-x}O_4$	-0.111	0.132	0.016	0.104
Dopant concentration (x)	-0.071	0.114	-0.071	0.091
Crystal size	**0.488**^{b}	0.124	0.304	0.101
Crystal size * crystal size	-0.433	0.233	**-0.440**^{b}	0.169
$Zn_xFe_{3-x}O_4$ * crystal size	0.024	0.203		
$Mn_xFe_{3-x}O_4$ * crystal size	-0.184	0.143		
$Mg_xFe_{3-x}O_4$ * crystal size	0.160	0.143		
x * size	0.063	0.101		
R^2	0.88		0.80	
Q^2	0.67		0.32	
Validity	0.38			
Reproducibility	0.99			

All responses were log transformed. ^{b}**Bold**: factors with the most influence on the given response. CI: confidence intervals.

REFERENCES

(1) Dadfar, S. M.; Roemhild, K.; Drude, N. I.; von Stillfried, S.; Knüchel, R.; Kiessling, F.; Lammers, T. Iron Oxide Nanoparticles: Diagnostic, Therapeutic and Theranostic Applications. *Adv. Drug Delivery Rev.* **2019**, *138*, 302–325.

(2) Sodipo, B. K.; Aziz, A. A. Recent Advances in Synthesis and Surface Modification of Superparamagnetic Iron Oxide Nanoparticles with Silica. *J. Magn. Magn. Mater.* **2016**, *416*, 275–291.
